# Crop wild relatives of legumes: evolutionary resources for climate-responsive pre-breeding

**DOI:** 10.3389/fpls.2026.1892793

**Published:** 2026-08-03

**Authors:** Dan Ioan Avasiloaiei, Mariana Calara, Petre Marian Brezeanu, Ioan Burzo, Creola Brezeanu

**Affiliations:** 1 Vegetable Research and Development Station, Bacău, Romania; 2 Gheorghe Ionescu-Şişeşti, The Academy of Agricultural and Forestry Sciences, Bucharest, Romania

**Keywords:** abiotic stress tolerance, climate resilience, genetic diversity, genomic selection, germplasm conservation, interspecific introgression, marker-assisted breeding, sustainable agriculture

## Abstract

Legume crops are increasingly exposed to a combination of abiotic stresses - including drought, heat, salinity and flooding - alongside mounting biotic pressures from pathogens and insect pests. Historical domestication and modern breeding practices have substantially narrowed the genetic base of cultivated legumes, constraining their adaptive potential and limiting yield stability under fluctuating and extreme environments. Crop wild relatives (CWRs) represent a vital reservoir of genetic variation, providing alleles that enhance physiological resilience, reproductive stability, stress-responsive signaling and symbiotic nitrogen fixation. Across major and minor legumes such as chickpea, lentil, lupin, pea, soybean, cowpea and common bean, their CWRs harbor both single-trait and multifaceted adaptive mechanisms, including robust root systems, efficient water and nutrient use, early phenology and resistance to emerging pests and diseases. These wild gene pools maintain functional diversity lost during domestication and constitute essential evolutionary resources for sustaining legume productivity while buffering cropping systems against climate variability. Landraces complement CWRs by offering pre-adapted, locally optimized phenotypes, providing alleles suited to specific agro-ecological niches. The integration of CWRs and landraces into breeding pipelines allows the capture of both cryptic and novel alleles governing complex polygenic traits. Modern breeding innovations - including high-throughput phenotyping, multi-omics platforms, genomic selection and genome editing - have tremendously enhanced the ability to exploit this diversity systematically. By minimizing linkage drag and overcoming reproductive constraints, these technologies accelerate the development of climate-resilient cultivars. Harnessing the combined evolutionary potential of CWRs and landraces with contemporary breeding approaches enables legumes to achieve greater productivity, yield stability and nutritional quality under dynamic environmental conditions, thereby reinforcing their role in sustainable agriculture and global food security.

## 1 Introduction

Palaeobotanical evidence indicates that the Fabaceae (legume) family originated during the Paleocene–early Eocene, approximately 60–56 million years ago, with subsequent diversification throughout the Cenozoic (Herendeen, 2001). During their evolutionary history, wild legume species were subjected to a wide range of biotic (e.g., pathogens, herbivores, symbiotic microorganisms) and abiotic (e.g., drought, temperature, soil nutrient availability) selective pressures. These environmental factors did not directly induce adaptive genetic changes but acted on naturally occurring genetic variation generated through mutation, recombination, gene flow, and genetic drift, resulting in populations adapted to diverse ecological conditions.

The transition from wild to domesticated legumes occurred much later, during the Neolithic agricultural revolution, approximately 12,000–10,000 years ago, when humans began cultivating and selectively propagating plants with desirable agronomic traits, including larger seeds, reduced seed shattering, synchronized maturation, and improved yield (Bohra et al., 2022). Early domestication was concentrated in several independent centres of agriculture, including the Fertile Crescent (Mesopotamia), the Nile Valley, and the Yellow River basin, where favourable environmental conditions supported crop cultivation.

Human influence on legume evolution has progressively intensified over time. Initial improvement relied on unconscious and later conscious phenotypic selection, followed by controlled hybridization and conventional breeding to combine desirable traits. More recently, molecular breeding, marker-assisted selection, genomic selection, and genetic engineering have enabled the precise introduction or modification of genes associated with disease resistance, abiotic stress tolerance, nutritional quality, and productivity. These successive stages of crop improvement have complemented natural evolutionary processes by accelerating the development of cultivars adapted to agricultural production systems. The evolution of plants has been and continues to be influenced by the great diversity of environmental factors that have driven their acclimatization or adaptation to the conditions present in different regions of the Earth: different terrestrial habitats, high or low humidity, low or high temperatures, strong or weak light intensity, nutrient deficiencies or excesses in the soil, and so on. Environmental factors that fall outside the optimal cultivation limits, along with pathogenic microorganisms, act as stressors that negatively affect the physiological and biochemical processes of plants.

Sensitivity to stress factors results from the combined effect of the plant’s receptor characteristics as number, sensitivity, and the properties of the stress factor itself: intensity, duration, etc. A stress factor must have high affinity for the receptor, saturate it, and persist for a sufficient period. Sudden exposure to intense, long-lasting stress has devastating effects on plants because it initiates biodegradation processes that lead to apoptosis that is, the death of cells and ultimately of the entire plant. Exposure to stress conditions slightly above the maximum tolerance limits can induce genetic modifications that persist for the duration of the stress and allow plant survival (acclimatization). If the stress factors act over a much longer period, defense responses may be epigenetically memorized and transmitted to offspring (adaptation). Meyer et al. (2012) note that approximately 2,500 species have undergone varying degrees of adaptation to cultivation conditions, of which 250 are considered fully adapted.

In this context, legumes represent a cornerstone of global food systems, contributing substantially to human nutrition, soil fertility and agroecological sustainability. Grain legumes (pulses), defined as the dry edible seeds of leguminous crops, are increasingly recognized for their central role in sustainable agriculture and dietary diversification (Acquah et al., 2021; Xavier et al., 2023). Their global importance was formally underscored by the United Nations’ declaration of 2016 as the International Year of Pulses, an initiative designed to highlight their contribution to food security, nutritional health and sustainable farming systems (Considine et al., 2017; Diedericks et al., 2020). Pulses provide high-quality plant protein (20–40%), essential amino acids, dietary fiber and micronutrients, serving as critical nutritional resources particularly in regions where access to animal protein is limited (Lal, 2017; Bessada et al., 2019; Teferra, 2021). Moreover, their capacity for biological nitrogen fixation enhances soil fertility and reduces dependency on synthetic fertilizers, thereby lowering production costs and environmental impacts (Guiguitant et al., 2020).

Despite their agronomic and nutritional significance, genetic improvement in many legume crops has lagged that of cereals, with relatively slow genetic gains reported across major species (Singh et al., 2022a). Yield stagnation, limited resilience to environmental stress and narrow adaptation ranges increasingly constrains legume productivity, particularly under rainfed and marginal conditions (Kumar et al., 2023). This stagnation is closely linked to the domestication bottleneck and subsequent breeding-driven genetic erosion that substantially reduced allelic diversity within cultivated gene pools (Singh et al., 2022a; Gupta and Bansal, 2023). Selection for uniform agronomic performance, seed size and reduced shattering inadvertently eliminated numerous adaptive alleles that were present in wild progenitors. Consequently, modern cultivars often lack sufficient genetic variation to respond effectively to intensifying abiotic stresses such as drought, salinity and extreme temperature fluctuations, as well as evolving pest and pathogen pressures (Pratap et al., 2021; Rajpal et al., 2023).

Climate change further exacerbates these limitations. Increased frequency of heat waves, irregular precipitation patterns, and soil degradation directly impair reproductive development, pollination success and yield stability in legumes. Concurrently, expanding pest populations and pathogen dynamics introduce additional biotic pressures (Singh et al., 2022a). The combination of climatic instability and narrow genetic bases restricts breeders’ ability to break yield plateaus and achieve the incremental gains required for future food security (Singh et al., 2022a). Addressing this challenge necessitates broadening the cultivated gene pool through systematic incorporation of novel alleles.

Crop wild relatives (CWRs) of legumes offer a powerful evolutionary resource to counteract domestication-induced genetic erosion. These undomesticated taxa, genetically related to cultivated species, have evolved under diverse and often harsh environmental conditions, retaining adaptive traits that were lost during domestication (Tripathi, 2019; Pratap et al., 2021). Wild gene pools frequently harbor alleles conferring tolerance to drought, salinity, and heat stress, as well as resistance to major diseases and insect pests (Zonneveld et al., 2020; Rajpal et al., 2023). The strategic exploitation of these reservoirs of variation is increasingly recognized as fundamental for developing climate-resilient cultivars capable of sustaining productivity under dynamic agroecological conditions (Gupta and Bansal, 2023; Rajpal et al., 2023).

However, despite their acknowledged importance, CWRs remain underutilized in mainstream legume breeding. Biological constraints such as ploidy differences, hybrid sterility and cross-compatibility barriers often limit gene flow between wild and cultivated taxa (Varshney et al., 2018; Rangan et al., 2023). Furthermore, the phenomenon of linkage drag - where undesirable genomic regions are co-introduced alongside beneficial alleles - reduces selection efficiency and may impose agronomic penalties, a challenge often described as part of the “cost of domestication” in reverse introgression strategies (Rangan et al., 2023). Compounding these biological obstacles is a historical scarcity of high-resolution phenotypic and genotypic data on economically important traits within wild germplasm collections (Zonneveld et al., 2020; Rajpal et al., 2023). Consequently, a comprehensive understanding of trait distribution across primary, secondary and tertiary gene pools remains incomplete, hindering systematic exploitation and conservation prioritization (Zonneveld et al., 2020).

The rapid advancement of genomic technologies has substantially enhanced the feasibility of exploiting CWRs in legume improvement programs. High-throughput sequencing, genome-wide association studies (GWAS), high-density linkage mapping, and pan-genomic analyses now enable the identification of adaptive genes, quantitative trait loci (QTLs), structural variants, and presence–absence polymorphisms that were previously inaccessible using conventional approaches (Varshney et al., 2018; Punia et al., 2022; Gupta and Bansal, 2023; Rajpal et al., 2023). These resources provide unprecedented resolution for characterizing genetic diversity within wild taxa and facilitate the precise tracking of beneficial alleles during introgression, thereby reducing the extent of donor genomic segments and minimizing linkage drag (Sharma et al., 2013; Singh et al., 2022a).

The translation of wild genetic diversity into breeding-ready materials increasingly relies on genomic-assisted pre-breeding approaches. Marker-assisted backcrossing, marker-assisted recurrent selection, and genomic selection enable the accumulation of favorable alleles governing complex quantitative traits such as stress adaptation, yield stability, and resource-use efficiency (Sharma et al., 2013; Gayacharan et al., 2023). The effectiveness of these strategies is further enhanced through the development of structured populations, including multi-parent advanced generation intercross (MAGIC) and nested association mapping (NAM) populations, which increase recombination frequency and facilitate the discovery of novel adaptive haplotypes (Singh et al., 2022a; Raina et al., 2023). When combined with speed breeding protocols capable of generating multiple generations annually, these approaches substantially accelerate genetic gain (Varshney et al., 2018; Singh et al., 2022a). The increasing availability of high-throughput phenotyping platforms is helping to overcome the long-standing phenotyping bottleneck in legume breeding. Integration of precision phenotyping with artificial intelligence-based predictive models and multi-omics datasets enables more accurate estimation of breeding values and improves the identification of superior donor accessions for climate-resilience traits (Rubiales et al., 2021; Gayacharan et al., 2023). These technologies provide a critical bridge between genomic information and field-level performance, strengthening selection efficiency across diverse environments. Genome editing technologies provide an additional layer of precision for deploying adaptive variation derived from CWRs. Targeted systems such as CRISPR/Cas permit site-specific modification of loci associated with stress adaptation and agronomic performance, circumventing many of the challenges associated with conventional introgression and linkage drag (Singh et al., 2022a). Functional validation studies in model and crop legumes have already demonstrated the feasibility of manipulating genes involved in root architecture, developmental plasticity, and abiotic stress responses, thereby accelerating the incorporation of beneficial variants into elite breeding backgrounds (Nadeem et al., 2023). Collectively, these innovations are transforming the utilization of wild germplasm and creating new opportunities for climate-responsive crop improvement.

**Figure 1:**
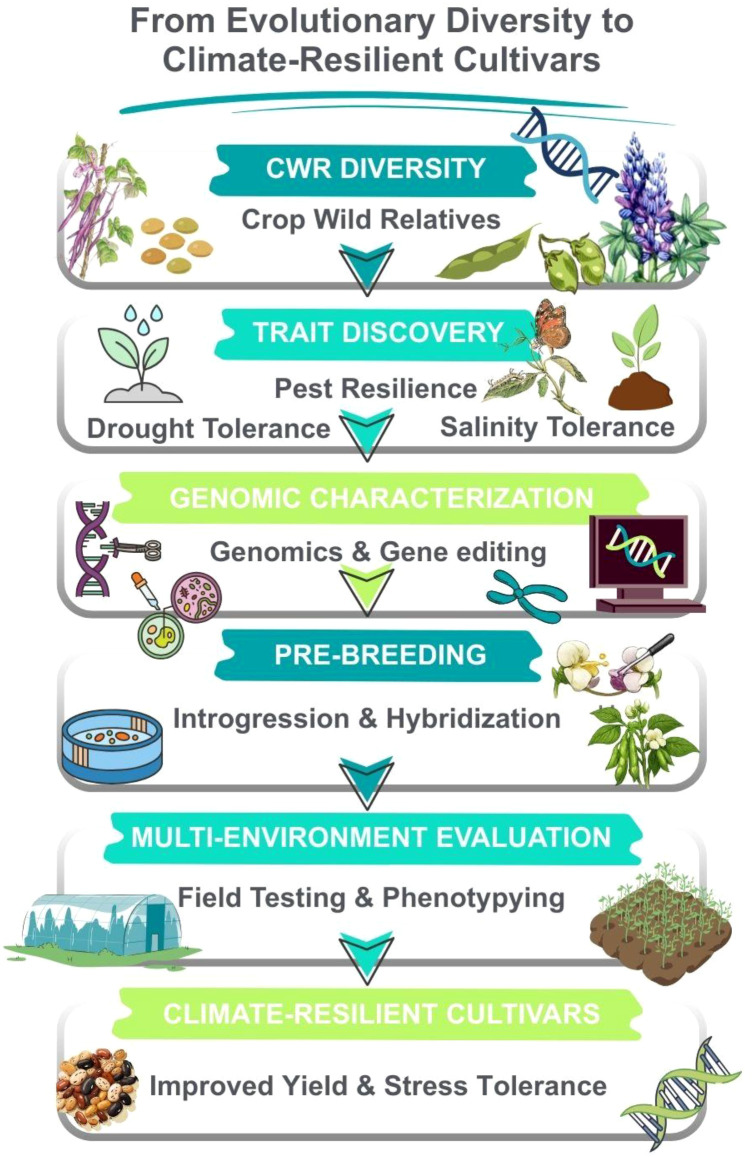
Rational steps from evolutionary diversity to climate resilient cultivars.

Beyond genetic considerations, strengthening legume productivity is also essential for achieving broader food system objectives. Global demand for pulses is projected to rise steadily, necessitating annual production growth exceeding 2% to meet consumption targets (Yadav et al., 2019). Yet production volatility, limited technological adoption, and policy constraints continue to restrict yield expansion in several major producing regions (Kumar and Raju, 2018; Kumar et al., 2023). Recognizing pulses not as “orphan crops” but as strategic components of sustainable food systems requires coordinated investment in research, breeding innovation and value-chain development (Adarsh et al., 2019; Robinson et al., 2019). Enhancing genetic resilience through CWR-based pre-breeding represents a foundational step in this transformation (**Figure 1**).

Although numerous studies have documented the value of CWRs as sources of stress tolerance and disease resistance, information remains fragmented across individual legume species, trait categories, and breeding platforms. Existing reviews have generally focused on single crops, specific stress factors, or isolated genomic technologies, limiting a comprehensive understanding of how evolutionary diversity can be systematically integrated into climate-responsive breeding pipelines. This review addresses this gap by providing a cross-species synthesis of legume CWRs, emphasizing their adaptive traits, gene-pool relationships, conservation status, and utility in pre-breeding. By linking evolutionary resources with emerging breeding technologies, this review presents a comprehensive framework for accelerating the development of climate-resilient legume cultivars under increasingly variable environmental conditions.

## 2 Climate change threats to legumes

### 2.1 Drought and heat stress

Legume production is increasingly constrained by the intensification of drought and heat episodes associated with global climate change (Krieg et al., 2024; Rajpal et al., 2023). Elevated temperatures and altered precipitation regimes impair key physiological processes, including photosynthesis, biomass accumulation, and reproductive development, ultimately leading to substantial yield penalties (Sita et al., 2017; Liu et al., 2019). Heat stress during flowering and pod set is particularly detrimental, frequently resulting in pollen sterility, reduced seed set, and shortened seed-filling duration (Sita et al., 2017; Liu et al., 2019). Under field conditions, drought and heat commonly co-occur, generating synergistic effects that exacerbate stomatal closure, limit carbon assimilation and intensify oxidative damage (Larmure and Munier‐Jolain, 2019; Sita et al., 2017).

Elevated temperatures represent a major constraint to legume productivity, affecting cellular homeostasis, photosynthesis, reproductive development, and yield stability. Heat stress perception involves osmosensory and signaling components, including histidine kinase receptors such as HK1, which activate downstream signaling cascades regulating stress-responsive genes and transcription factors (Franco-Zorrilla et al., 2014). These molecular responses promote the synthesis of heat shock proteins (HSPs), which function as molecular chaperones by stabilizing protein structure, preventing aggregation, and facilitating refolding of damaged proteins. The degree of thermal tolerance varies among plant species, with heat-sensitive plants generally tolerating temperatures up to 35–40 °C, heat-tolerant species surviving temperatures approaching 50 °C, and thermophilic species enduring temperatures exceeding 50 °C and, in some cases, reaching 60–65 °C (Źróbek-Sokolnik, 2011). Moderate temperature increases of 5–10 °C above the optimum may be tolerated, whereas increases of 12–15 °C typically induce physiological stress responses (Leone et al., 2003). In legumes, the accumulation of HSPs has been associated with enhanced thermotolerance; for example, pea plants synthesize low-molecular-weight HSPs (17.9–18.1 kDa) at temperatures between 30 and 42 °C (DeRocher et al., 1991), while heat-tolerant bean cultivars induce a broader spectrum of HSPs than sensitive genotypes under elevated soil temperatures (Michiels et al., 1994).

Drought adaptation in legumes relies on a coordinated network of physiological and molecular responses that maintain cellular hydration and metabolic activity under water deficit. Water limitation is perceived through osmosensory pathways involving HK1, leading to activation of stress-responsive signaling and abscisic acid (ABA) biosynthesis. ABA plays a central regulatory role by inducing stomatal closure, thereby reducing transpirational water loss, and by activating downstream pathways involved in stress protection. These include the synthesis of late embryogenesis abundant (LEA) proteins and dehydrins, which stabilize cellular structures during dehydration, as well as the accumulation of compatible osmolytes such as soluble sugars, proline, organic acids, and polyols that contribute to osmotic adjustment. Consistent with these mechanisms, drought-stressed pea plants accumulated soluble carbohydrates at levels 1.5–7-fold higher than well-watered controls (Sanchez et al., 1998), with subsequent studies reporting 2.8–5.1-fold increases in soluble sugars within the epicotyl under water deficit conditions (Sanchez et al., 2004). Collectively, ABA-mediated signaling, protective protein accumulation, and osmotic adjustment constitute the principal mechanisms by which legumes maintain physiological function during drought stress.

Beyond direct impacts on carbon metabolism, these stresses critically disrupt biological nitrogen fixation (BNF), a cornerstone of legume productivity (Vadez et al., 2011; Krieg et al., 2024). Abiotic stress can impair the host plant, the rhizobial partner, or their symbiotic interaction, collectively reducing nitrogen-fixing efficiency (Renzi et al., 2022). Elevated soil temperatures inhibit nitrogenase activity and modulate nodulation-related gene expression, thereby constraining nodule formation and function (Peng et al., 2025; Dwivedi et al., 2014). Structural and metabolic deterioration of nodules, including reduced nodule number, weight and ureide transport, has been documented under combined heat and drought stress (Goyal et al., 2021; Chaudhary et al., 2022). Moreover, high root-zone temperatures suppress rhizobial survival and infection processes, with growth inhibition reported between 40–47 °C in soybean and common bean (Alemayehu et al., 2018; Sita et al., 2017).

Identifying stress-resilient germplasm and compatible, temperature-tolerant rhizobial strains is therefore critical for sustaining BNF under climate variability (Dwivedi et al., 2014; Sita et al., 2017). Integrating such adaptive traits into pre-breeding pipelines represents a strategic pathway toward climate-responsive legume improvement.

### 2.2 Salinity and soil degradation

The expansion of agriculture into marginal lands increasingly affected by salinity and soil degradation represents a major constraint to legume productivity under climate change. Salinity imposes both osmotic and ionic stress, restricting water uptake, nutrient acquisition, and overall plant vigor, while simultaneously disrupting the legume–rhizobium symbiosis that underpins biological nitrogen fixation (Anjum, 2016; Renzi et al., 2022). High concentrations of Na^+^ and Cl⁻ in the rhizosphere impair nodulation, reduce nodule biomass and inhibit nitrogenase activity, thereby diminishing nitrogen inputs essential for yield stability (Peña and Pueyo, 2011; Wekesa et al., 2022). The sensitivity of this process is compounded by salinity-induced reductions in leghemoglobin content and nodule respiration, which constrain oxygen supply to bacteroid, and limit ATP regeneration required for ammonia assimilation (Shree et al., 2022; Aridhi et al., 2020).

Saline conditions also alter rhizobial survival and infectivity, with differential tolerance reported among genera, where Rhizobium and Sinorhizobium often outperform more sensitive Bradyrhizobium strains (Goyal et al., 2021). At the whole-plant level, ionic imbalance disrupts Ca²^+^ and K^+^ homeostasis, depresses photosynthetic efficiency, and accelerates oxidative stress, collectively reducing biomass accumulation and yield (Ali et al., 2023; Bouzroud et al., 2023). Soil degradation processes, including erosion and acidification linked to intensive fertilizer use, further exacerbate these constraints and are projected to intensify with climate-driven land-use pressures (Bellabarba et al., 2023; Oparah et al., 2023).

Salt stress increases the osmotic pressure of the cytosol due to the accumulation of high concentrations of salts. Sensitive legume species (such as common bean) tolerate salinity levels of 0–3 dS/m, while moderately tolerant species (such as soybean) tolerate 6–8 dS/m. Osmotic stress caused by salinity is perceived by a histidine kinase (HK1), which activates transporter proteins that exclude sodium from the cytoplasm by sequestering it into vacuoles or the apoplast. The SOS1 gene encodes a plasma membrane Na^+^/H^+^ antiporter that removes excess Na^+^ from the cytoplasm and accumulates it in the apoplast or in the roots. The NHX gene encodes a tonoplast Na^+^/H^+^ antiporter that transports sodium from the cytoplasm into the vacuole. The histidine-kinase transporter HKT1 mediates the transport of sodium and potassium from the apoplast into the symplast. Because it has low affinity for Na^+^, its transport of sodium decreases under toxic concentrations, limiting Na^+^ entry into cells (Burzo, 2014).

Salinity tolerance in legumes is highly genotype-dependent and reflects substantial variation in the regulation and functional efficiency of salt-responsive genes. Although tolerant and susceptible genotypes often activate similar stress-response pathways, they differ markedly in the expression of genes controlling ion transport, osmotic adjustment, antioxidant defense, and photosynthetic stability (Kaashyap et al., 2018; Khan et al., 2023). In chickpea, contrasting genotypes exhibit differential expression of Na^+^/H^+^ antiporters, vacuolar ATPases, and transcription factors, leading to distinct capacities for ionic homeostasis under saline conditions (Khan et al., 2023). Similarly, allelic variation at major loci such as *GmSALT3* and *GmSALT18* contributes to differential Na^+^ exclusion and tissue ion compartmentalization among soybean genotypes (Chen et al., 2023). These findings demonstrate that not all genotypes within traditionally salt-sensitive legume species are equally susceptible and highlight the extensive phenotypic and molecular diversity present within cultivated germplasm and wild relatives (HanumanthaRao et al., 2016; Roorkiwal et al., 2020). The occurrence of contrasting salinity tolerance phenotypes within individual legume species underscores the significance of intraspecific diversity as a source of adaptive genetic variation, providing valuable donor germplasm for breeding programs aimed at enhancing salt tolerance (Kaashyap et al., 2017; Ali et al., 2022).

Adaptive responses involve complex regulatory networks controlling ion transport, osmolyte biosynthesis, and antioxidant defense systems (Araújo et al., 2014; Noor et al., 2024). However, cultivated legumes remain largely sensitive to salinity, underscoring the importance of exploiting CWRs as reservoirs of alleles conferring enhanced ionic tolerance and symbiotic stability under degraded soil conditions (Danial and Basset, 2024).

### 2.3 Climate-driven shifts in pest and pathogen dynamics

Climate change is reshaping host–pathogen and plant–insect interactions, intensifying biotic pressures on both cultivated legumes and their wild relatives (Rubiales et al., 2021; Vadez et al., 2011). Rising temperatures and altered precipitation regimes facilitate the geographic expansion of pests and pathogens, accelerate infection cycles, and enhance overwintering survival, thereby increasing epidemic risk (Vadez et al., 2011; Pourkheirandish et al., 2020). Empirical evidence links extreme weather events to heightened disease severity, including dry root rot in chickpea and Phytophthora blight in pigeonpea under heat and moisture stress conditions (Dwivedi et al., 2013), as well as Alternaria blight outbreaks triggered by untimely rainfall in semi-arid systems (Sharma et al., 2013). Projected global temperature increases of 2–3 °C are expected to further destabilize species physiology and phenology, fostering the emergence of new virulent strains and biotypes (Rajpal et al., 2023; Pratap et al., 2021).

Modern cultivars, shaped by domestication bottlenecks and intensive selection, often harbor a narrow genetic base that limits the availability of durable resistance alleles (Varshney et al., 2019; Singh et al., 2022a). This vulnerability is exacerbated by the rapid evolutionary rates of phytopathogens, whose short life cycles enable swift adaptation to resistant hosts (Son and Park, 2022; Roy et al., 2023). CWRs therefore constitute indispensable reservoirs of resistance genes for broadening the genetic base and enhancing resilience (Zonneveld et al., 2020; Maxted and Brehm, 2023). Strategic introgression and pre-breeding can generate novel recombinants for deployment in mainstream breeding pipelines (Pratap et al., 2021).

Climate-driven shifts in pest and pathogen distribution increasingly threaten legume productivity, highlighting the need to exploit CWRs as reservoirs of resistance alleles lost during domestication (Pratap et al., 2021; Zonneveld et al., 2020). Wild tepary bean (*Phaseolus acutifolius*), for example, exhibits resistance to *Xanthomonas* and rust pathogens, demonstrating the potential of CWRs for broadening the genetic basis of disease resistance (Smýkal et al., 2014). These responses are mediated by regulatory networks involving nucleotide-binding leucine-rich repeat (NLR) genes, receptor-like kinases (RLKs), salicylic acid signaling, and defense-related transcription factors that coordinate pathogen recognition and immune activation (Dodds and Rathjen, 2010; Almeida et al., 2014; Muñoz et al., 2017). In addition, β-conglutins identified in lupin (*Lupinus* spp.) contribute to innate defense against fungal and oomycete pathogens, providing an alternative source of durable resistance (Rubiales et al., 2018). Moreover, resistance gene homologs identified across Fabaceae provide a rich source of candidate loci for enhancing disease resistance in cultivated legumes (Varshney et al., 2009).

Resistance loci and their associated regulatory networks are now being resolved at increasingly higher resolution, improving our understanding of plant–pathogen interactions and supporting the deployment of durable resistance under rapidly evolving pathogen pressures (Mousavi‐Derazmahalleh et al., 2018a; Parihar et al., 2022; Burdon and Zhan, 2020; Huang et al., 2023a).These tools, combined with marker-assisted selection, resistance gene enrichment, and genome editing, facilitate the efficient introgression and deployment of adaptive alleles into elite germplasm (Duc et al., 2014). However, environmental modulation of R gene–mediated immunity, particularly under elevated temperatures, can compromise resistance durability, underscoring the need to integrate evolutionary and ecological perspectives into breeding strategies (Tsai et al., 2022; Miedaner and Juroszek, 2021). Consequently, pyramiding of multiple NLR genes with complementary specificities remains a key strategy for achieving durable, broad-spectrum resistance in changing climates (Dangl et al., 2013).

### 2.4 Interactive abiotic–biotic stress networks under climate change

**Figure 2:**
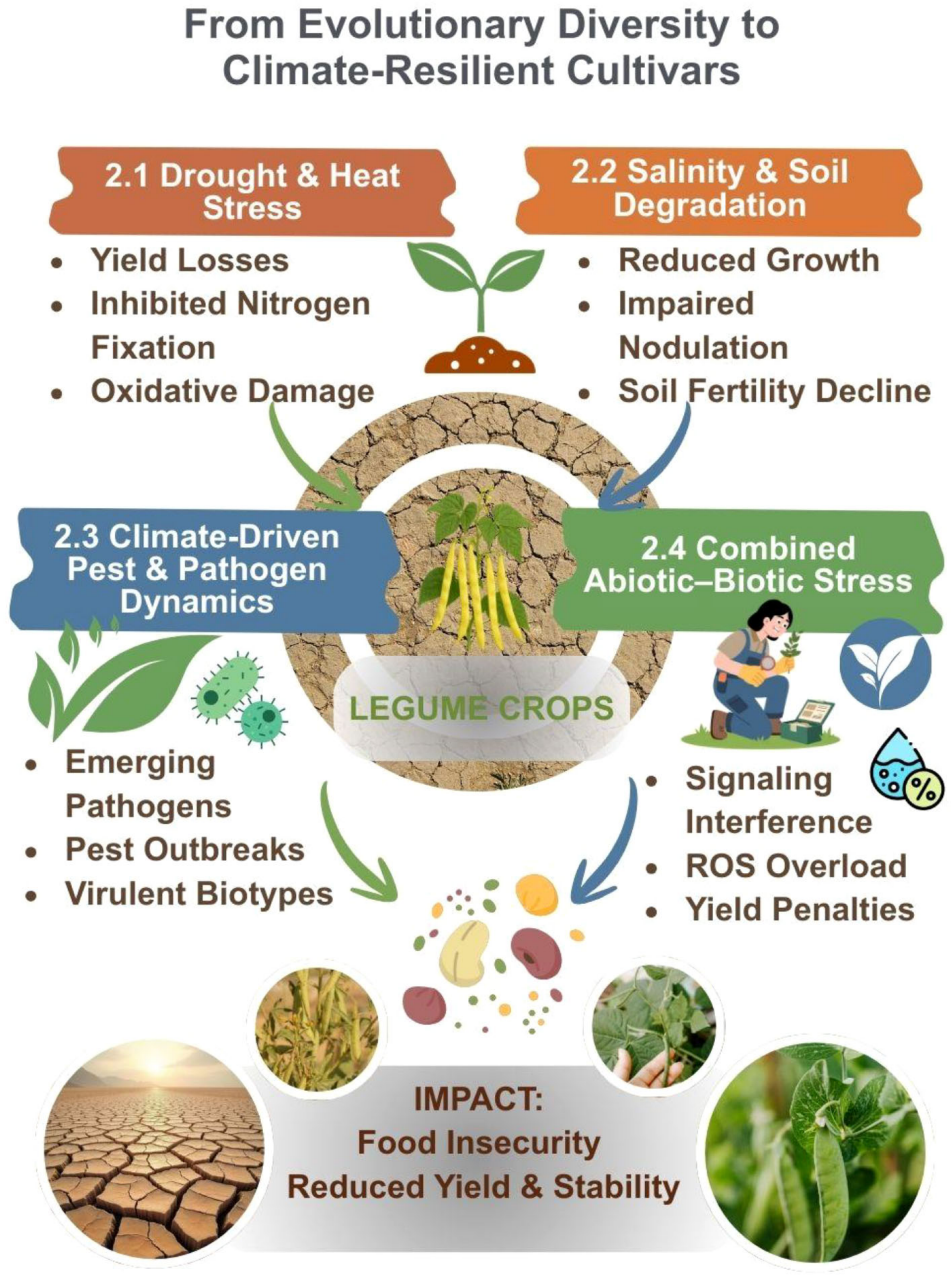
Multifactorial stress regimes, from evolutionary diversity to climate resilient cultivars.

Climate change is amplifying the convergence of abiotic and biotic stresses, generating complex environmental scenarios that challenge legume productivity and global food security (Krieg et al., 2024; Rajpal et al., 2023). Heat and drought episodes increasingly coincide with outbreaks of pathogens and insect pests, reshaping plant–pest interactions and often enhancing host susceptibility while diminishing competitive ability against weeds (Pandey et al., 2017; Zonneveld et al., 2020). Evidence indicates that simultaneous stresses typically impose greater yield penalties than individual factors acting in isolation (**Figure 2**), reflecting synergistic physiological disruption across developmental stages (Pandey et al., 2017).

At the molecular level, plants deploy intricate signaling networks to perceive and respond to environmental cues; however, concurrent activation of stress pathways frequently results in signaling interference, trade-offs, or prioritization of one response over another (Mota et al., 2021). Phytohormonal crosstalk, transcription factor cascades, and kinase-mediated signaling modules integrate abiotic and biotic stimuli, yet these shared regulatory nodes can produce antagonistic or synergistic outputs that ultimately determine plant performance (Rejeb et al., 2014; Kissoudis et al., 2014). Elevated reactive oxygen species accumulation under combined stresses further intensifies cellular damage, often overwhelming antioxidant defenses and accelerating productivity losses (Leonetti et al., 2023; Araújo et al., 2014).

Importantly, transcriptomic analyses reveal that combined stress responses are not predictable from single-stress datasets, as demonstrated in wild *Arachis* species, underscoring the distinct genetic architecture underlying stress interactions (Mota et al., 2021). CWRs of legumes, adapted to heterogeneous and frequently harsh environments, represent invaluable models for dissecting these complex adaptive mechanisms (Adamik et al., 2023; Balestrini et al., 2022). Advancing breeding for climate resilience therefore requires a strategic shift toward identifying shared regulatory hubs and pyramiding compatible resistance components to sustain yield stability under multifactorial stress regimes (Kissoudis et al., 2014; Mota et al., 2021).

## 3 Genetic erosion and the need for broader diversity

### 3.1 Domestication bottlenecks and genetic narrowing in legumes

The domestication of legume crops has been accompanied by pronounced genetic erosion, largely driven by strong selection for a limited set of agronomic traits such as increased seed size, uniform maturation, and reduced pod shattering (Singh et al., 2022a). These selection pressures, combined with founder effects and repeated reliance on a narrow pool of elite breeding lines, have substantially reduced allelic diversity within cultivated gene pools (Pratap et al., 2021; Singh et al., 2022a). As a result, many modern legume cultivars exhibit limited adaptive potential, contributing to the relatively slow genetic gains observed in legume improvement programs, particularly under rapidly changing climatic conditions (Singh et al., 2022a).

Domestication and subsequent breeding have narrowed the genetic base of many legume crops, leaving numerous adaptive alleles absent from modern cultivars. CWRs provide access to this untapped diversity, including traits associated with tolerance to abiotic stresses and resistance to emerging pests and diseases (Pratap et al., 2021; Dwivedi et al., 2017). However, their utilization has been constrained by biological barriers, linkage drag, and insufficient phenotypic and genomic characterization (Zonneveld et al., 2020; Ambika et al., 2022). The exploitation of secondary and tertiary gene pools has become increasingly feasible, allowing the systematic identification and transfer of beneficial alleles that were previously difficult to access through conventional breeding strategies (Razzaq et al., 2021; Vikram et al., 2024). Expanding the cultivated gene pool through these approaches is therefore essential to counteract domestication-driven genetic narrowing and to build resilient, climate-responsive legume cultivars for future agricultural systems (Kapazoglou et al., 2023; Dwivedi et al., 2023).

### 3.2 Modern breeding limitations and the imperative for diverse germplasm

The recurrent use of elite lines in legume breeding has led to a pronounced narrowing of the genetic base, constraining adaptive plasticity and resilience to biotic and abiotic stresses (Rubiales et al., 2021; Singh et al., 2022a). Prioritization of readily adapted germplasm over exotic or wild relatives exacerbates genetic bottlenecks, leading to the re-circulation of identical genotypes and reduced deployment of valuable alleles lost during domestication (Sharma et al., 2013; Zonneveld et al., 2020; Pratap et al., 2021). Biological barriers such as linkage drag and cross-incompatibility further limit the incorporation of beneficial alleles from secondary and tertiary gene pools, necessitating specialized techniques like embryo rescue, mentor pollination and hormone applications to bypass pre- and post-fertilization constraints (Pratap et al., 2021). Complementary strategies including speed breeding, genome editing, and haplotype-based selection can compress breeding cycles and enhance the expression of desirable alleles (Baloğlu et al., 2022; Rangan et al., 2023). Establishing cost-effective genotyping platforms and global phenotyping networks, alongside digital genebanks linking genomic and phenotypic data, is essential to facilitate the access to diverse germplasm and sustain genetic gains, thereby enabling climate-resilient legume improvement (Dwivedi et al., 2023; Smýkal et al., 2014; Cortés and Barnaby, 2023).

### 3.3 Climate-driven vulnerability and the strategic deployment of wild diversity

Domestication bottlenecks and the recurrent use of elite breeding lines have markedly reduced genetic variability in cultivated legumes, heightening their susceptibility to climatic instability and emerging pathogens (Singh et al., 2022a). This constrained genetic base limits the effective introgression of adaptive alleles from wild germplasm, particularly those conferring tolerance to drought, salinity and complex biotic stresses (Zonneveld et al., 2020; Singh et al., 2022a). Paradoxically, CWRs - critical reservoirs of evolutionary resilience - are themselves threatened by habitat loss, with more than half considered endangered, underscoring the urgency of their conservation and use (Civantos-Gómez et al., 2022; Rajpal et al., 2023).

Although linkage drag and limited trait characterization have historically hindered wild introgression, advances in functional genomics, genome-wide association studies, and high-density mapping now enable precise identification of favorable alleles while minimizing undesirable genomic segments (Varshney et al., 2018; Rajpal et al., 2023; Zonneveld et al., 2020). The deployment of multi-parent populations such as MAGIC and NAM, coupled with genomics-assisted and haplotype-based breeding, enhances allelic recombination and accelerates pre-breeding pipelines (Pratap et al., 2021; Singh et al., 2022a). Integration of speed breeding, gene editing, and high-throughput genotyping further compresses generation intervals and facilitates targeted allele fixation without compromising elite backgrounds (Baloğlu et al., 2022; Kulkarni et al., 2018). A tiered strategy combining alien introgression, precision breeding, and data-driven selection is therefore pivotal to restore genetic gain and secure climate-resilient legume production systems (Rangan et al., 2023; Raina et al., 2023). **Table 1** presents the summary of the key issues, consequences and modern strategies, tools identified in literature for assessing limitation of the approaches discussed above.

**Table 1:** Summary of genetic erosion, breeding constraints and strategies for leveraging crop wild relatives (CWRs) in legumes.

Category	Key issues	Consequences	Modern strategies/tools	Representative references
Domestication bottlenecks	Selection for limited agronomic traits (seed size, uniformity, pod shattering); founder effects; reliance on elite lines	Narrowed allelic diversity; reduced adaptive potential; slow genetic gains	Introgression from secondary/tertiary gene pools; pan-genome construction; pre-breeding programs	Singh et al., 2022a; Pratap et al., 2021; Dwivedi et al., 2017; Razzaq et al., 2021; Vikram et al., 2024
Modern breeding limitations	Recurrent use of elite lines; prioritization of adapted germplasm; linkage drag; cross-incompatibility	Reduced deployment of valuable alleles; limited adaptive plasticity; vulnerability to biotic/abiotic stresses	Embryo rescue, mentor pollination, hormone treatments; high-throughput phenotyping; genomics-assisted breeding; genomic selection; speed breeding; genome editing; haplotype-based selection	Rubiales et al., 2021; Singh et al., 2022a; Sharma et al., 2013; Varshney et al., 2018; Baloğlu et al., 2022; Rangan et al., 2023
Climate-driven vulnerability	Narrow genetic base; endangered CWRs due to habitat loss; insufficient trait characterization	Heightened susceptibility to drought, salinity, and emerging pathogens; stagnation of genetic gain	Multi-parent populations (MAGIC, NAM); GWAS and high-density mapping; speed breeding; gene editing; high-throughput genotyping; digital genebanks; integrated alien introgression pipelines	Singh et al., 2022a; Zonneveld et al., 2020; Civantos-Gómez et al., 2022; Rajpal et al., 2023; Kulkarni et al., 2018; Raina et al., 2023
Strategic CWR deployment	Underutilization of wild alleles; barriers to introgression	Loss of resilience alleles; slower adaptation to environmental change	Precision pre-breeding; alien gene introgression; tiered breeding programs; integration of conventional breeding with bioinformatics, phenotyping, and omics; high-throughput selection	Singh et al., 2022a; Pratap et al., 2021; Gayacharan et al., 2023; Baloğlu et al., 2022; Rangan et al., 2023

## 4 Crop wild relatives as sources of climate-adaptive traits

### 4.1 Definition, gene pools and conservation status

The genetic diversity distributed across primary, secondary, and tertiary gene pools offers valuable opportunities for broadening the adaptive capacity of cultivated legumes through the incorporation of stress-resilience and disease-resistance traits (Smýkal et al., 2014; Cortés and Barnaby, 2023). Despite their recognized value, *in situ* conservation of CWRs remains limited, reflecting a disconnect between academic recommendations and practical management due to insufficient coordination between plant genetic resource communities and protected area managers (Smýkal et al., 2014; Bönisch et al., 2025). Many owners are unaware of the presence of valuable germplasm on lands under their stewardship, highlighting the need for targeted policy engagement and incentives to support conservation (Bönisch et al., 2025; Smýkal et al., 2014). Regions such as the western Fertile Crescent, identified as a hotspot for temperate legume diversity, remain largely unprotected despite documented genetic erosion (Rouichi et al., 2024).

To optimize conservation, innovative approaches including species distribution modeling and gap analysis are increasingly applied to identify priority populations, map diversity hotspots, and guide both *in situ* and *ex situ* strategies (Smýkal et al., 2017; Kägi et al., 2023; Vincent et al., 2019). Nevertheless, global *ex situ* collections remain inadequate, with up to 95% of priority CWRs underrepresented in genebanks (Raggi et al., 2022). Integrating phenotypic and genotypic characterization with ecogeographic sampling and digital genebanks can enhance access to adaptive diversity for pre-breeding programs (Salgotra and Chauhan, 2023; Álvarez et al., 2015). Multidisciplinary collaboration across conservation, genomics and breeding sectors is essential to translate CWRs diversity into climate-resilient cultivars, supporting sustainable agricultural production and food security under ongoing environmental change (Tirnaz et al., 2022; Maxted and Brehm, 2023; Pathirana and Carimi, 2022).

**Figure 3:**
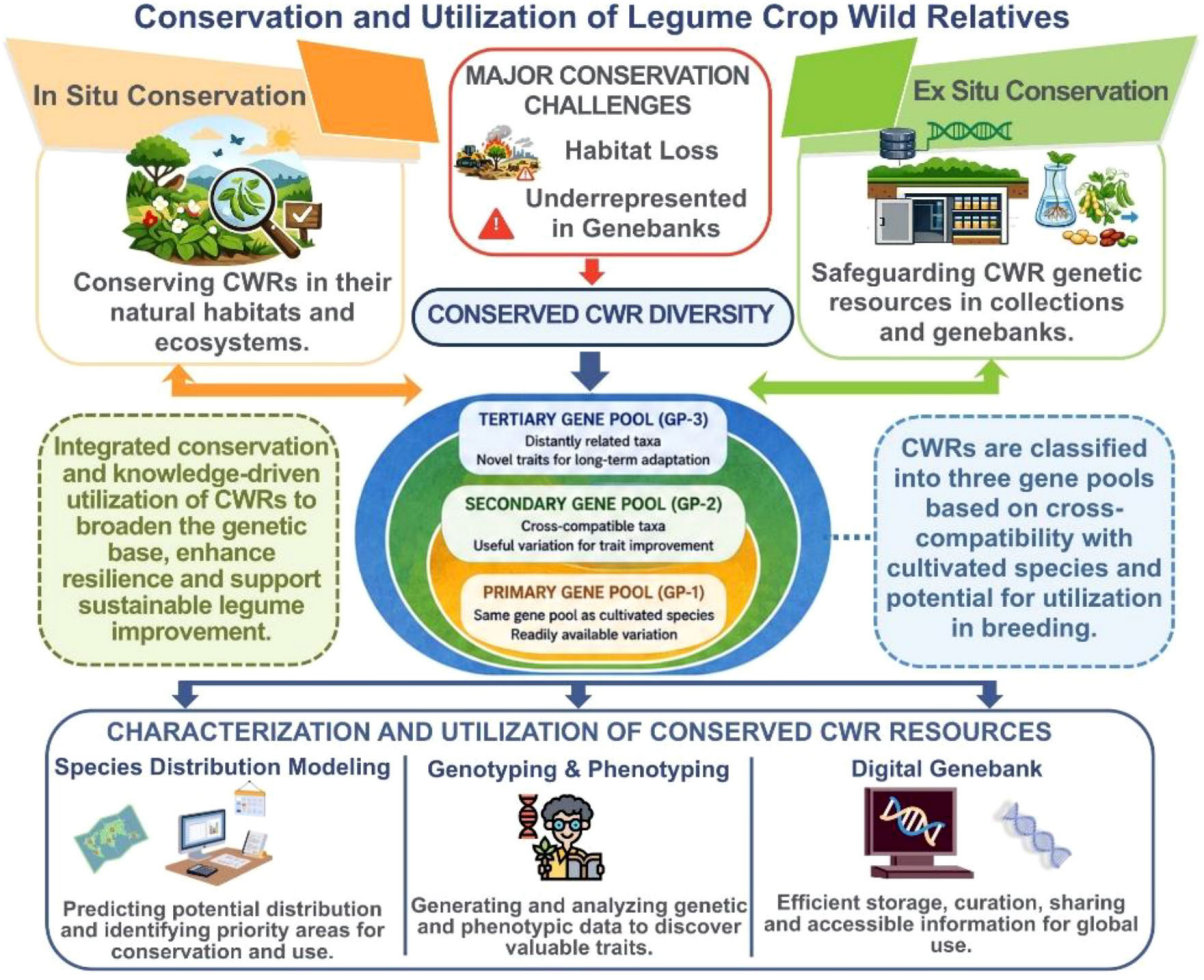
Conservation and utilization of legumes CWRs.

Wild relatives, landraces and locally adapted traditional populations contain vital genetic diversity that must be collected, conserved and effectively utilized in breeding programs (**Figure 3**). Despite significant efforts in recent years, these resources are increasingly threatened by the effects of climate change as well as by various human-induced pressures, including intensive agricultural practices and frequent socio-economic changes. Another paradox arises from the gap between the importance of these species for food security and the still limited knowledge about their existing diversity, particularly how it is distributed and how it can be more widely used to improve cultivated crops.

### 4.2 Climate-relevant adaptive traits

The strategic integration of CWRs into breeding pipelines offers a robust pathway to overcome the genetic constraints imposed by domestication and to enhance resilience in legume crops under climate variability. Within this context, wild *Vigna* species constitute a particularly valuable genetic reservoir, encompassing broad adaptive diversity across more than 80 taxa. These species harbor alleles associated with tolerance to heat, drought, and salinity, as well as resistance to key pests and pathogens. Notably, several *Vigna* taxa exhibit adaptive traits such as early flowering and rapid life cycle completion, enabling escape from extreme temperatures exceeding 40 °C and terminal water deficits. Species including *Vigna trilobata*, *V. stipulacea*, and *V. subramaniana* further demonstrate the capacity to withstand combined abiotic and biotic stresses, reflecting the presence of multifunctional or stacked adaptive mechanisms (Zonneveld et al., 2020).

Comparable potential is evident within wild *Lens* and *Cicer* gene pools, which contribute valuable alleles for both stress tolerance and agronomic performance. For instance, *Lens lamottei* provides strong resistance to Stemphylium blight, while *Lens odemensis* confers resistance to the Sitona weevil. In addition, *Lens ervoides* contributes traits related to phenology, biomass accumulation, and growth habit, all of which are critical for adaptation to marginal environments. Similarly, wild *Cicer* species such as *C. judaicum* serve as important sources of resistance to *Ascochyta blight* (Pratap et al., 2021; Rajpal et al., 2023).

Beyond stress resistance, these wild relatives retain the capacity to sustain symbiotic nitrogen fixation under suboptimal environmental conditions, thereby supporting productivity in low-input systems. This functional resilience provides an opportunity to enhance nutrient-use efficiency and reduce dependence on synthetic fertilizers, aligning legume improvement strategies with the goals of sustainable and climate-resilient agriculture (Pratap et al., 2021).

**Figure 4:**
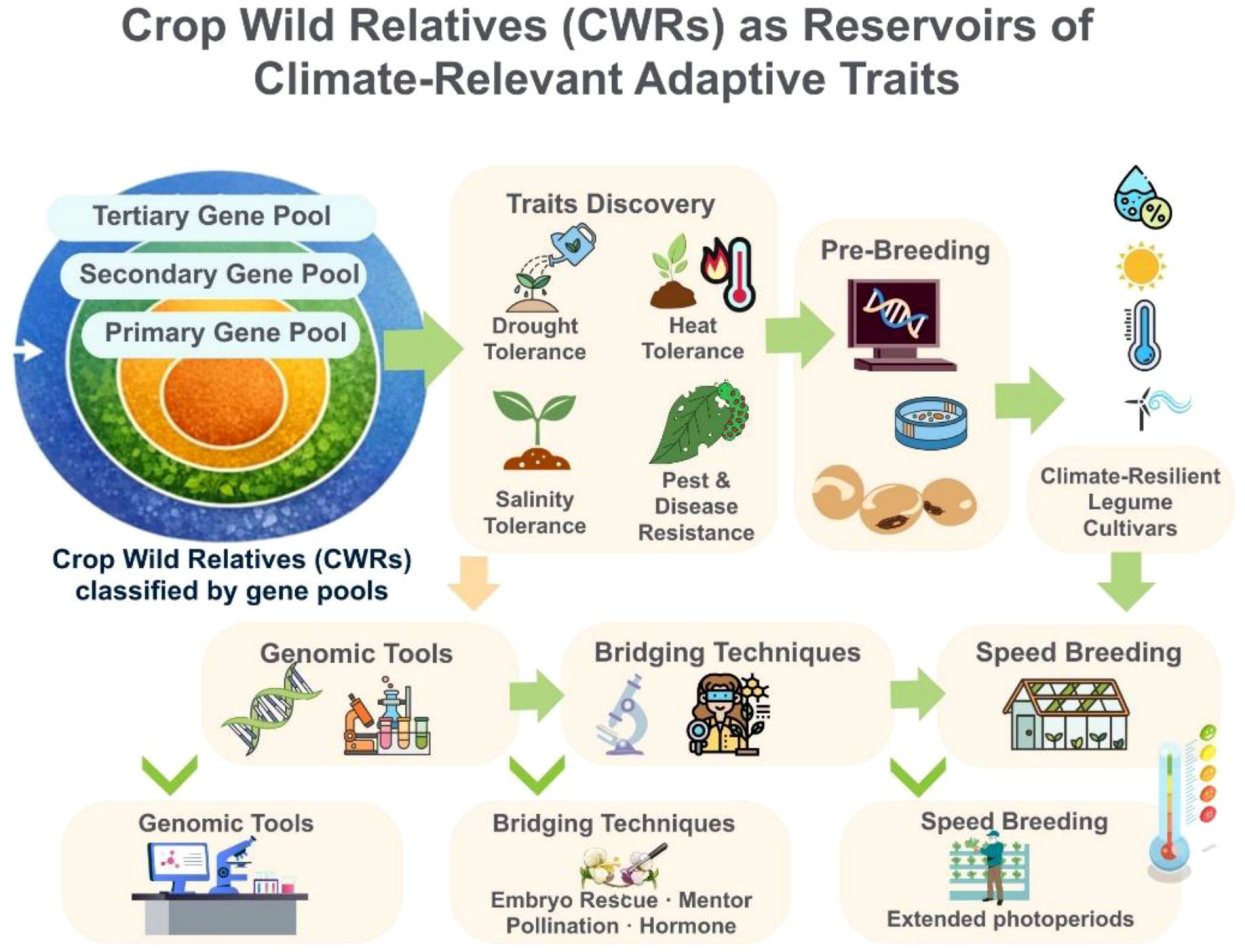
CWRs as reservoirs of climate – relevant adaptative traits.

Integrating CWRs diversity into breeding programs requires advanced phenomics and genomics platforms (**Figure 4**). Multi-parent populations, such as MAGIC and NAM, enable the dissection of complex traits and the creation of elite recombinants, facilitating the detection of quantitative trait loci associated with stress tolerance, yield, and quality (Rajpal et al., 2023; Singh et al., 2022a). Marker-assisted selection, genomic selection, and high-throughput genotyping accelerate the precise introgression of desirable alleles while minimizing linkage drag, thereby expanding the adaptive potential of cultivated germplasm (Dwivedi et al., 2023; Samal et al., 2023; Gayacharan et al., 2023). Genomics-assisted pre-breeding further allows for efficient tracking of alien alleles and the combination of rare genes from wild gene pools, addressing the stagnation in genetic gain caused by limited primary gene pool diversity (Sharma et al., 2013; Singh et al., 2022a).

Overcoming reproductive barriers, such as ploidy differences in *Arachis* or hybridization incompatibilities in wild chickpea, necessitates specialized techniques, including embryo rescue, mentor pollination, and hormone-assisted fertilization, enabling distant hybridization and the incorporation of adaptive traits (Pratap et al., 2021; Jiménez-López et al., 2020; Varshney et al., 2018). The assembly of high-density genetic maps, pan- and super-pan genomes, and whole-genome sequencing of CWRs has further modernized breeding pipelines, enabling high-resolution QTL mapping, haplotype identification, and precision introgression of adaptive traits (Rajpal et al., 2023; Gayacharan et al., 2023; Pandey et al., 2016).

Finally, integrating speed breeding and high-throughput phenotyping with genomic-assisted strategies compresses breeding cycles, enhances selection intensity and allows rapid fixation of favorable alleles from wild relatives, ultimately accelerating the development of climate-resilient, high-yielding and nutritionally superior legume cultivars (Singh et al., 2022; Kumar et al., 2024; Naqvi et al., 2022; Dwivedi et al., 2023). Collectively, the systematic incorporation of CWR-derived adaptive traits into breeding programs represents an indispensable approach to construct resilient legume production systems capable of sustaining stable yields under increasingly erratic environmental conditions.

### 4.3 Utilization of crop wild relatives for trait enhancement in major legumes

Evidence from chickpea, soybean, common bean, lentil, lupin, pea, and cowpea demonstrates that introgression from wild germplasm broadens the adaptive variation available to breeding programs and enhances resilience to multiple stressors (Gupta and Bansal, 2023; Kapazoglou et al., 2023). Alleles associated with heat and drought adaptation have been identified in *Cicer reticulatum*, whereas wild *Cicer* species contribute resistance to major diseases. Similarly, *Cajanus platycarpus, C. volubilis*, and *C. cajanifolius* have provided photoperiod insensitivity and extra-early flowering in pigeonpea, facilitating adaptation to changing agroecological conditions (Krieg et al., 2024; Singh et al., 2022).

Wild Lens taxa harbor allelic variation controlling yield stability, stress tolerance, and adaptive phenology that is largely absent from cultivated germplasm (Rajpal et al., 2023). Resistance introgression has also been achieved through the transfer of Ascochyta blight resistance from *Pisum fulvum* and recessive pea weevil resistance genes (*pwr1, pwr2, pwr3*), while *P. sativum* ssp. *elatius* provides resistance to *Fusarium wilt* and *Mycosphaerella pinodes* (Tayeh et al., 2015; Singh et al., 2022). In common bean, *Phaseolus acutifolius* contributes resistance to bacterial blight and rust together with allelic diversity in drought-responsive transcription factors such as ASR and DREB (Smýkal et al., 2014). Perennial wild *Glycine* species and wild *Vigna* relatives similarly represent important sources of resistance, yield stability, and stress-resilience traits for soybean and cowpea improvement, respectively (Singh et al., 2022).

The effective utilization of these resources remains constrained by hybridization barriers, linkage drag, low recombination frequencies, and heterogeneous crossover landscapes between wild and cultivated genomes (Dwivedi et al., 2023; Hübner and Kantar, 2021). Embryo rescue, mentor pollination, growth regulator treatments, and genomics-assisted breeding approaches facilitate the transfer and fixation of beneficial alleles, while functional genomics, genomic selection, and machine-learning tools improve the identification of superior haplotypes for marker-assisted introgression (Sharma et al., 2019; Pratap et al., 2021; Flint-Garcia et al., 2023; Rangan et al., 2023). Together, these advances strengthen the integration of CWRs into breeding pipelines aimed at counteracting genetic erosion and enhancing climate resilience in legume crops (Rajpal et al., 2023; Singh et al., 2022).

#### 4.3.1 Common bean (*Phaseolus* spp.)

The evolutionary differentiation of the Andean and Mesoamerican gene pools in common bean (Phaseolus vulgaris L.) has resulted in contrasting genetic architectures and phenotypic responses to variation in water availability. Under non-stress conditions, pods per plant contribute directly to yield; however, under drought, high pod numbers frequently translate into poor seed filling, requiring gene pool–specific selection indices that prioritize seed weight, pod partitioning index (PPI), and harvest index (HI) (Asfaw et al., 2017). The high heritability and genetic advance of PPI and HI confirm the importance of efficient photosynthate remobilization under terminal drought, a mechanism supported by the identification of a stable, major QTL on linkage group Pv07 associated with yield maintenance (Terán et al., 2019). Nonetheless, introgression of drought tolerance between Andean and Mesoamerican races - particularly from Durango or Mesoamerican backgrounds into large-seeded Andean types - has often resulted in agronomically inferior progeny due to negative inter-gene pool interactions (Hamabwe et al., 2023).

To circumvent these constraints, breeding efforts increasingly exploit adaptive variation from wild *Phaseolus* taxa occupying diverse eco-geographic regions. Wild tepary bean (*Phaseolus acutifolius*) has emerged as a key reservoir of drought and heat tolerance, as well as resistance to common bacterial blight, angular leaf spot, *Xanthomonas*, and rust pathogens (Smýkal et al., 2014; Assefa et al., 2019; Wu et al., 2022). Its adaptation to arid environments is underpinned by morphological and physiological traits including tolerance to temperatures above 32 °C, precise stomatal regulation, dehydration avoidance, fine and deeply penetrating root systems with high hydraulic conductivity, early maturity, and strong remobilization capacity (Beebe et al., 2013; Polanía et al., 2016, 2022; Cruz et al., 2023). Distinct drought strategies have been characterized within *P. acutifolius*, ranging from water-saving genotypes exhibiting early stomatal closure and reduced leaf area (e.g., line G 40001) to water-spending types maintaining higher stomatal conductance and biomass accumulation before terminal stress (Polanía et al., 2016, 2022).

Additional wild relatives provide complementary adaptive traits. *Phaseolus parvifolius* has facilitated hybridization as a bridging species, enhancing male gametic diversity and increasing interspecific hybrid recovery between *P. vulgaris* and *P. acutifolius* (Dwivedi et al., 2023). *Phaseolus coccineus* contributes thick root systems capable of penetrating compacted soils, offering alternative drought avoidance mechanisms (Beebe et al., 2013). Molecular analyses have revealed adaptive allelic variation at stress-responsive loci such as *Dreb2B* and *ERECTA* in *P. acutifolius* and *P. parvifolius*, with sequence polymorphisms correlating with environmental drought indices (Cortés and Blair, 2018; Dwivedi et al., 2023).

Overcoming reproductive barriers between primary and tertiary gene pools has required advanced techniques, including embryo rescue and bridging lines (Smýkal et al., 2014; Cruz et al., 2023). Emerging tools such as long-read sequencing, genomic prediction, machine learning, and detection of introgression footprints now enable more precise dissection of complex polygenic traits while minimizing linkage drag (Chacón-Sánchez et al., 2021; Mutari et al., 2023). However, phenotyping bottlenecks and genotype-by-environment interactions remain critical challenges (Cortés et al., 2022). According to Bitocchi et al. (2017) *Phaseolus* represents an exceptional model for investigating crop evolution and the convergent phenotypic trajectories associated with domestication. Within this genus of approximately 70 species, five (*P. vulgaris, P. coccineus, P. dumosus, P. acutifolius*, and *P. lunatus*) underwent independent domestication. Notably, *P. vulgaris* and *P. lunatus* possess wild populations in both Mesoamerica and South America, where at least two geographically isolated domestication events occurred. Collectively, these patterns reflect a minimum of seven independent domestication episodes, offering a rare framework for dissecting the genetic architecture of domestication both across species and within species-level gene pools. The genus is further distinguished by features that enhance its value for evolutionary research: a relatively recent divergence accompanied by strong genomic collinearity and synteny, substantial variation in breeding systems and life-history strategies, and broad ecological adaptability, both within native ranges and following global dissemination. In *P. vulgaris*, the worldwide spread of cultivation has facilitated secondary contact between the Mesoamerican and Andean gene pools, enabling spontaneous hybridization and the emergence of novel genetic and phenotypic diversity. Combined with extensive *ex situ* and *in situ* conservation efforts, these attributes make *Phaseolus* a powerful system for elucidating the genetic basis of adaptation and for guiding the development of improved varieties capable of addressing future challenges in climate resilience, food security, and sustainable agriculture. The strategic conservation and characterization of wild *Phaseolus* germplasm collections, including extensive *P. acutifolius* accessions and interspecific lines, therefore represent foundational pillars for climate-responsive pre-breeding in common bean (Rodriguez et al., 2022).

#### 4.3.2 Chickpea (*Cicer* spp.)

The genus *Cicer* comprises 44 species, 9 annual and 35 perennials (Harlan and de Wet, 1971; van der Maesen, 1987) of which *C. arietinum* (cultivated chickpea) is the only domesticated form. Its primary gene pool includes *C. arietinum* and its direct wild progenitor *C. reticulatum*, which crosses readily. The secondary gene pool consists of *C. echinospermum*, capable of hybridization with reduced fertility, while the remaining annual and perennial species form the tertiary gene pool, requiring advanced methods for gene transfer. Wild *Cicer* species are predominantly distributed across West Asia and North Africa, from Turkey to Ethiopia and from Pakistan to Morocco. Four major centers of chickpea genetic diversity have been identified: the Mediterranean, Central Asia, the Near East, and India, with Ethiopia serving as a secondary center. Species occupy a wide ecological range, from sea level to over 5,000 m in the Himalayas. While *C. arietinum* has a broad global distribution, key wild relatives such as *C. bijugum*, *C. echinospermum*, and *C. reticulatum* are restricted mainly to Turkey, Syria, and Iraq, with other species occurring in the Levant, Afghanistan, and the trans-Himalayan region. These areas represent hotspots of *Cicer* diversity (Chand Jha et al., 2024) Cultivated chickpea (*Cicer arietinum* L.) exhibits an exceptionally narrow genetic base, with diversity levels estimated to be nearly 100-fold lower than those of its wild progenitors due to domestication bottlenecks and founder effects (Varshney et al., 2018; Von Wettberg et al., 2018). This erosion has constrained adaptive potential to abiotic stresses such as drought, salinity, heat, and cold, as well as to major pathogens and insect pests (Pratap et al., 2021; Varshney et al., 2019). Consequently, wild *Cicer* taxa constitute indispensable reservoirs of allelic variation for climate-responsive pre-breeding.

Within the primary gene pool, *Cicer reticulatum* - the direct progenitor - is readily crossable with the cultigen and has provided resistance to cyst nematode (e.g., accession ILWC119), cold tolerance, and resistance to wilt, foot rot, root rot, and Botrytis gray mold (Pratap et al., 2021; Sharma et al., 2013). The secondary gene pool, particularly *Cicer echinospermum*, has been instrumental in transferring genes for multiple biotic and abiotic stresses and in generating early maturing, high-yielding introgression lines (Buhariwalla et al., 2005; Singh et al., 2022b). Additional wild species—including *Cicer bijugum*, *Cicer judaicum*, *Cicer pinnatifidum*, and *Cicer cuneatum*—have demonstrated resistance to *Fusarium wilt* and *Botrytis* gray mold, broadening the spectrum of exploitable defense mechanisms (Jha et al., 2020; Pratap et al., 2021). Tolerance to drought and heat has been documented across several *Cicer* species, underscoring their ecological breadth (Pratap et al., 2021).

Historically, introgression from these wild relatives was constrained by linkage drag and crossing barriers (Roorkiwal et al., 2014; Zonneveld et al., 2020). However, genomics-assisted breeding, haplotype-based approaches, and specialized population designs are now enabling efficient transfer of quantitative trait loci while purging deleterious alleles (Pratap et al., 2021; Singh et al., 2022b). High-density genotyping platforms such as the Axiom^®^ CicerSNP Array, together with draft genome assemblies and genome-wide association studies, have facilitated fine mapping of resistance loci and candidate genes, including *Ca_14301* (NB-ARC domain), *CaFeSOD*, *CaS13like*, *CaNTAQ1*, and receptor-like kinases associated with Fusarium wilt resistance (Roorkiwal et al., 2020; Mannur et al., 2018; Alsamman et al., 2024). Marker-assisted backcrossing has successfully deployed these loci into elite cultivars, exemplified by improved lines such as Super Annigeri 1 and JG 74315-14 derived from donors like WR 315 (Jha et al., 2020).

Integration of wild introgression populations involving *C. reticulatum* and *C. echinospermum* with multi-omics and precise phenotyping platforms is redefining chickpea pre-breeding, accelerating the recovery of adaptive alleles lost during domestication and strengthening resilience to climate variability (Roorkiwal et al., 2020; Singh and Sood, 2024; Singh et al., 2022b).

#### 4.3.3 Lentil (*Lens* spp.)

Genetic improvement in lentil (*Lens culinaris* ssp. *culinaris*) has been constrained by a narrow-domesticated gene pool, reflecting historical bottlenecks and limited exploitation of CWRs (Zonneveld et al., 2020; Pratap et al., 2021). High-throughput sequencing combined with GWAS and population genomic analyses has revealed adaptive alleles, stress-associated QTLs, and candidate genes in wild Lens taxa, providing molecular targets for the introgression of climate-resilience traits into cultivated lentil (Gupta and Bansal, 2023; Rajpal et al., 2023).

Phylogenomic analyses have delineated four gene pools within *Lens*: primary, secondary, tertiary, and quaternary (Dadu et al., 2021). The primary and secondary pools include the cultivated species and its progenitor, *Lens culinaris* ssp. *orientalis*, a key source of alleles for cold tolerance and resistance to *Ascochyta blight, Stemphylium blight, Fusarium wilt*, and rust (Rajpal et al., 2023). Tertiary gene pool species, notably *Lens ervoides* and *Lens nigricans*, provide resistance to anthracnose, powdery mildew, rust, and *Orobanche* spp., as well as adaptive variation for drought tolerance (Singh et al., 2014; Pratap et al., 2021). Additional taxa such as *Lens odemensis* have shown drought resilience, broadening the spectrum of exploitable traits (Pratap et al., 2021).

Among these, *L. ervoides* has emerged as a particularly rich reservoir of fungal disease resistance. Composite interval mapping in recombinant inbred populations identified 11 QTLs for resistance to *Colletotrichum lentis* and three for *Stemphylium botryosum*, spanning genomic regions enriched in NBS-LRR proteins, chitinases, defensins, peroxidases, and ABC transporters (Bhadauria et al., 2017; Rajpal et al., 2023). Resistant accessions such as L01–827A and IG 72815 exhibit broad-spectrum resistance to anthracnose*, Ascochyta blight*, and *Stemphylium blight* (Bhadauria et al., 2017). Rust and powdery mildew resistance have also been identified in *L. nigricans* ILWL 191 and *L. ervoides* ILWL 91 and ILWL 269 (Singh et al., 2014).

Beyond biotic stress, wild *Lens* taxa harbor alleles for salinity, heat, cold, and boron tolerance, alongside improved micronutrient content, photo-insensitivity, and early maturity (Dadu et al., 2021; Rajpal et al., 2023; Singh et al., 2022). Pre-breeding efforts at ICARDA using *L. orientalis* and *L. ervoides* derivatives have achieved yield gains exceeding 40%, demonstrating the translational value of wild diversity in lentil improvement (Rajpal et al., 2023; Singh et al., 2022). Lentil CWRs remain an underutilized source of novel alleles and adaptive traits, particularly those conferring resistance to emerging biotic and abiotic stresses driven by climate change. Despite their potential, wild *Lens* species have been only partially explored, and substantial genetic and genomic diversity remains untapped. Expanding the characterization and use of these wild relatives is essential for broadening the genetic base of cultivated lentil. This includes assessing their diversity, understanding their genomic resources, and integrating beneficial traits through introgression (Dasgupta and Barman, 2024).

#### 4.3.4 Pea (*Pisum* spp.)

The improvement of cultivated pea (*Pisum sativum* L.) for winter hardiness and drought adaptation remains constrained by frost injury and limited root plasticity under variable soil moisture (Duc et al., 2014; Beji et al., 2020). Wild *Pisum* relatives provide critical reservoirs of adaptive traits to address these limitations. Notably, *Pisum fulvum* exhibits rapid and deep root development, enhanced lateral root density, and steeper root angles, conferring superior drought avoidance and water acquisition under arid conditions (Araújo et al., 2014; Bakır et al., 2025). In contrast, *Pisum sativum* subsp. *elatius* (syn. *P. elatius*) demonstrates physiological mechanisms underpinning freezing tolerance, reflecting its winter growth habit and capacity to overwinter vegetatively (Smýkal et al., 2017; Araújo et al., 2014). Broader germplasm screenings have identified frost-tolerant accessions, including ATC 104 and ATC 377, and numerous resilient genotypes across diverse collections (Gayacharan et al., 2023).

Genetic dissection of frost tolerance has revealed major QTLs on linkage groups III, V, and VI, stable across environments and explaining substantial phenotypic variance (Beji et al., 2020). Genome-wide association studies further identified 62 SNPs across six linkage groups and 50 candidate genes associated with frost damage (Bhat et al., 2022). Central to these loci are C-repeat binding factor (CBF/DREB) transcription factors, particularly within the LD block VI.2 region, where AP2/ERF genes colocalize with freezing tolerance QTLs (Tayeh et al., 2013; Beji et al., 2020). Comparative genomics highlights strong synteny with the Mt-FTQTL6 region on chromosome 6 of *Medicago truncatula*, which harbors tandem CBF clusters, suggesting conserved mechanisms of cold adaptation across legumes (Araújo et al., 2014; Perdomo et al., 2022). Similar macrosynteny with faba bean reinforces a shared evolutionary origin of frost tolerance loci within the Papilionoideae (Perdomo et al., 2022).

Despite challenges of linkage drag and crossing barriers (Zonneveld et al., 2020), high-density gene-based maps, pan-genomic sequencing, and genomic selection models now facilitate precise introgression of polygenic traits such as frost tolerance and root architecture (Michel et al., 2019; Sallam et al., 2022). By integrating genome-wide markers with major QTLs, breeders can pyramid favorable alleles from *P. fulvum* and *P. sativum* subsp. *elatius* into elite cultivars, accelerating the development of climate-resilient pea varieties adapted to freezing stress and water-limited environments (Rubiales et al., 2021; Gupta and Bansal, 2023).

#### 4.3.5 Lupin (*Lupinus* spp.)

CWRs of lupins constitute an important reservoir of adaptive traits that can support climate-responsive breeding in grain legumes. Wild progenitors of the genus *Lupinus* have evolved in nutrient-poor and environmentally marginal habitats, particularly across the Mediterranean basin, where strong selective pressures have shaped efficient nutrient acquisition strategies and tolerance to edaphic stresses (Mousavi‐Derazmahalleh et al., 2018b; Msaddak et al., 2023). Species such as *Lupinus angustifolius* (narrow-leafed or blue lupin), *L. albus* (white lupin), and *L. luteus* (yellow lupin) exemplify this adaptive potential, displaying substantial variation in phenology, water-use efficiency, and biomass production that contributes to resilience in harsh agroecological environments (Renzi et al., 2022; Valente et al., 2023).

Among these, *L. angustifolius* is particularly adapted to infertile, sandy, and acidic soils, where its root system architecture supports efficient nutrient acquisition and enables cultivation under conditions unsuitable for many crops (Mousavi‐Derazmahalleh et al., 2018b). A key adaptive mechanism is the formation of specialized cluster roots that enhance phosphorus mobilization from poorly soluble soil pools through rhizosphere acidification (Lucas et al., 2015; Pantigoso et al., 2020). Similarly, *L. albus* exhibits exceptional phosphorus acquisition efficiency, even in the absence of mycorrhizal associations, due to its capacity to release carboxylate-rich exudates that mobilize recalcitrant phosphates (Xu et al., 2020; Robles-Aguilar et al., 2020). These mechanisms, coupled with biological nitrogen fixation, contribute to improved soil fertility and reduced reliance on synthetic fertilizers in sustainable cropping systems (Książkiewicz et al., 2017; Rychel et al., 2020).

Wild and semi-domesticated lupins also exhibit adaptive responses to water limitation. Certain *L. luteus* ecotypes maintain higher relative leaf water content under severe drought conditions, reflecting physiological strategies that enhance resilience in Mediterranean climates characterized by seasonal drought (Smýkal et al., 2014). These traits are often associated with root plasticity, including increased root:shoot ratios and enhanced exploration of deeper soil layers, which facilitate water and nutrient uptake (Pantigoso et al., 2020; Griffiths et al., 2021).

Advances in molecular and genomic tools have significantly accelerated the exploitation of lupin diversity for pre-breeding. Transcriptomic studies have revealed regulatory networks associated with drought tolerance and nutrient acquisition in lupin roots (Burchardt et al., 2024; Pueyo et al., 2021), while genetic analyses in *L. angustifolius* have identified loci controlling phenology and environmental adaptation (Taylor et al., 2021; Mousavi‐Derazmahalleh et al., 2018b). The availability of high-quality reference genomes, particularly for *L. albus*, together with genotyping-by-sequencing and genome-wide association studies, now facilitates the identification of markers linked to phosphorus use efficiency, nitrogen fixation, and yield stability (Hufnagel et al., 2020; Dwivedi et al., 2023; Schwertfirm et al., 2024). These resources provide a foundation for genomic selection and marker-assisted breeding aimed at developing lupin cultivars adapted to marginal soils and future climate conditions (Nadeem et al., 2023; Snapp, 2020).

#### 4.3.6 Cowpea (*Vigna* spp.)

Cowpea (*Vigna unguiculata* [L.] Walp.) is a cornerstone grain legume in sub-Saharan Africa, valued for its protein-rich seed and intrinsic tolerance to drought and heat (Carvalho et al., 2017). This resilience is linked to deep rooting, maintenance of symbiotic nitrogen fixation under water deficit, and physiological mechanisms that sustain productivity under thermal stress (Abebe and Alemayehu, 2022; Carvalho et al., 2017). Nevertheless, cultivated gene pools harbor limited diversity for complex stress-adaptive traits, prompting renewed emphasis on wild *Vigna* relatives as reservoirs of novel alleles (De Ron et al., 2018; Zonneveld et al., 2020).

Wild taxa distributed across humid to arid ecogeographic gradients capture broader adaptive spectra than domesticated lines (Iseki et al., 2018). Screening of cultivated and wild accessions revealed significant variation for tolerance to both drought and excess moisture stress, traits increasingly relevant under projected climate variability in West Africa (Iseki and Olaleye, 2025). However, comprehensive phenotyping indicates that fewer than 30% of evaluated *Vigna* accessions exhibit strong abiotic stress resilience, reflecting a historical bias toward biotic resistance in wild populations (Zonneveld et al., 2020). Notably, *Vigna aridicola* demonstrates pronounced dehydration tolerance in permanently hot climates, whereas *Vigna vexillata* var. *ovata* combines dehydration and salinity resilience in seasonally dry habitats (Zonneveld et al., 2020). Many taxa within the subgenus *Vigna* (gene pools C and D), occupying marginal soils or flood-prone environments, remain insufficiently characterized for stress tolerance (Zonneveld et al., 2020).

Key drought-adaptive traits in wild *Vigna* include enhanced root biomass, deeper non-superficial rooting systems with greater hydraulic conductivity, xylem vessel architecture, root suberization, regulated stomatal conductance, and reduced leaf hydraulic conductance (Iseki et al., 2018; Zonneveld et al., 2020; Polanía et al., 2022). These features support water conservation and sustained photosynthetic activity, complemented in some genotypes by osmotic adjustment and abscisic acid-mediated stomatal regulation (Araújo et al., 2014). Importantly, wild accessions often maintain pod set and seed filling under stress through efficient photosynthate remobilization (Araújo et al., 2014).

Given projections of prolonged and erratic drought episodes, breeding strategies must prioritize introgression of constitutive tolerance mechanisms, such as deep rooting and heat-stable photosynthesis, over sole reliance on phenological escape (Iseki et al., 2018; Zonneveld et al., 2020). Integration of high-throughput phenomics, including infrared thermography, with iterative, environment-specific trait deployment will be essential to translate wild adaptive diversity into climate-resilient cowpea cultivars (Nair et al., 2019; Beebe et al., 2013).

#### 4.3.7 Soybean (*Glycine* spp.)

Wild soybean (*Glycine soja* Sieb. & Zucc.), the direct progenitor of cultivated soybean (*Glycine max*), together with perennial taxa within the subgenus *Glycine*, represents a major reservoir of adaptive variation for waterlogging and hypoxia tolerance. Comparative analyses indicate that wild *Glycine* germplasm retains functional alleles that have been partially lost during domestication, particularly those associated with root system performance and stress resilience (Sharmin et al., 2021).

Genetic dissection of these traits reveals predominantly polygenic architecture. A key quantitative trait locus, qWT_Gm03 on chromosome 3, has been linked to enhanced root system plasticity under oxygen-limited conditions (Wang et al., 2024). In addition, multiple QTLs located on chromosomes 11–14 regulate root length, diameter, and surface area, all of which are critical for maintaining root functionality during hypoxia (Wang et al., 2024). Genome-wide association studies have further identified loci such as QTN13 on chromosome 13, associated with germination, seedling vigor, and membrane stability under flooding, with candidate genes including *Glyma.13g248000* carrying functional polymorphisms (Sharmin et al., 2021). Additional SNPs (e.g., Gm_08_11971416 and Gm_08_46239716) and differentially expressed genes such as *Glyma.01G231200* and *Glyma.08G083300* underscore the value of wild alleles for marker-assisted pyramiding strategies (Sharmin et al., 2021).

At the physiological level, tolerance is mediated by coordinated anatomical and metabolic adjustments. The formation of aerenchyma and adventitious roots enhances internal oxygen transport and compensates for impaired primary root systems under waterlogged conditions (Xu et al., 2022a). Concurrently, metabolic reprogramming toward anaerobic fermentation sustains cellular energy supply, with increased activity of pyruvate decarboxylase and alcohol dehydrogenase enabling ATP production under oxygen deprivation (Lin et al., 2019; Gedam et al., 2023; Zhang et al., 2025). These responses are regulated by group VII ethylene response factors within the AP2/ERF transcription factor family, which function as central regulators of hypoxia sensing via the N-end rule pathway (Wang et al., 2020; De Ollas et al., 2021).

In contrast to rice, soybean lacks major-effect loci such as SUBMERGENCE-1 and SNORKEL, necessitating the pyramiding of multiple minor-effect QTLs to achieve stable tolerance (Nakayama et al., 2017; Jhan et al., 2023). Therefore, effective pre-breeding strategies must integrate wild *Glycine* diversity with genomic selection frameworks capable of capturing genotype-by-environment interactions and enabling precise phenotyping of root architecture and associated physiological traits (Sharmin et al., 2021; Zhang et al., 2024a).

**Table 2** summarizes representative CWRs as key evolutionary resources for climate-responsive pre-breeding in major legume species, emphasizing their adaptive traits and relevance for stress resilience.

**Table 2:** Crop wild relatives as evolutionary resources for climate-responsive pre-breeding in major legumes.

Crop	Wild taxa (gene pool)	Key adaptive traits	Genetic architecture/key loci	Pre-breeding tools & achievements	Key references
Common bean *(Phaseo-lus vulgaris)*	*P. acutifolius* (tertiary); *P. parvifolius; P. coccineus*	Heat (>32 °C) and drought tolerance; deep fine roots; high hydraulic conductivity; stomatal regulation; remobilization efficiency; resistance to bacterial blight, rust, angular leaf spot	Major QTL on Pv07 for yield under drought; adaptive allelic variation at Dreb2B, ERECTA; inter-gene pool epistasis (Andean × Mesoamerican)	Embryo rescue; bridging species (*P. parvifolius*); genomic prediction; long-read sequencing; introgression footprint detection	Asfaw et al., 2017; Terán et al., 2019; Smýkal et al., 2014; Dwivedi et al., 2023
Chickpea (*Cicer arieti-num*)	*C. reticulatum* (primary); *C. echinospermum* (secondary); C. bijugum, C. judaicum, C. pinnatifidum, C. cuneatum	Drought, heat, salinity, cold tolerance; resistance to Fusarium wilt, Botrytis, cyst nematode	Candidate genes: Ca_14301 (NB-ARC), CaFeSOD, RLKs; multi-locus QTL introgression; narrow domesticated diversity (~100-fold lower)	Axiom^®^ CicerSNP Array; haplotype-based breeding; marker-assisted backcrossing (e.g., Super Annigeri 1)	Varshney et al., 2019; Pratap et al., 2021; Roorkiwal et al., 2020
Lentil *(Lens culinaris* ssp. culinaris*)*	*L. orientalis* (primary); *L. ervoides*, *L. nigricans* (tertiary); *L. odemensis*	Resistance to anthracnose, rust, powdery mildew; drought, salinity, boron tolerance; micronutrient enrichment	11 QTLs for Colletotrichum lentis; NBS-LRR clusters; pan-genomic resources	ICARDA pre-breeding; >40% yield gain via wild introgression; high-density maps and transcriptomics	Bhadauria et al., 2017; Rajpal et al., 2023
Pea *(Pisum sativum)*	*P. fulvum; P. sativum* ssp. elatius	Deep rooting; frost tolerance; resistance to *Ascochyta blight*, pea weevil, *Fusarium wilt*	Major frost QTLs (LG III, V, VI); CBF/DREB clusters; synteny with Medicago truncatula Mt-FTQTL6	Genomic selection; pan-genomics; pyramiding frost tolerance alleles	Beji et al., 2020; Tayeh et al., 2013
Lupin *(Lupinus* spp.*)*	*L. angustifolius*; *L. albus*; *L. luteus*	Adaptation to acidic, sandy, nutrient-poor soils; cluster root formation; enhanced phosphorus mobilization via citrate and malate exudation; high phosphorus-use efficiency without mycorrhizae; symbiotic nitrogen fixation; drought tolerance via root plasticity and higher leaf relative water content	Loci controlling phenology and environmental adaptation in *L. angustifolius*; genes regulating cluster root formation and P acquisition; genetic linkage of alkaloid content and growth traits (*iucundus*, *tardus*)	Genotyping-by-sequencing; GWAS; high-quality reference genome for *L. albus*; genomic selection and marker-assisted breeding for P-use efficiency and nitrogen fixation	Mousavi‐Derazmahalleh et al., 2018b; Hufnagel et al., 2020; Dwivedi et al., 2023; Nadeem et al., 2023; Pantigoso et al., 2020
Cowpea *(Vigna unguiculata)*	*V. aridicola; V. vexillata* var. *ovata; subgenus Vigna (gene pools C & D)*	Deep rooting; hydraulic conductivity; xylem architecture; dehydration & salinity tolerance; osmotic adjustment; ABA-mediated stomatal regulation	<30% accessions show strong abiotic resilience; polygenic drought tolerance	Infrared phenomics; iterative environment-specific trait deployment	Iseki et al., 2018; Zonneveld et al., 2020
Soybean *(Glycine max)*	G. soja (primary progenitor); perennial Glycine spp. (subgenus Glycine)	Waterlogging and hypoxia tolerance; aerenchyma formation; adventitious rooting; enhanced anaerobic fermentation; root system plasticity	QWT_Gm03 (Chr 3); QTLs (Chr 11–14) controlling root traits; QTN13 (Chr 13); candidate genes (*Glyma.13g248000*, *Glyma.01G231200*, *Glyma.08G083300*); AP2/ERF group VII regulation; absence of SUB1/SNORKEL orthologs	GWAS; SNP marker development (e.g., Gm_08_11971416, Gm_08_46239716); genomic selection integrating G×E; physiological phenotyping of root trait	Wang et al., 2024; Sharmin et al., 2021; Lin et al., 2019; Gedam et al., 2023; Zhang et al., 2025

## 5 Landraces: locally adapted genetic resources

### 5.1 Evolutionary adaptation under farmer selection

Landraces represent dynamic, heterogeneous populations shaped by the interplay of natural selection and farmer-driven preferences, serving as invaluable reservoirs of genetic diversity for breeding programs (Suso et al., 2016). Unlike modern cultivars, these traditional varieties maintain complex mixtures of homozygous and heterozygous lines, reflecting centuries of adaptation to local agro-ecological conditions (De Ron et al., 2018; Plekhanova et al., 2017). This evolutionary process results in populations with high intra-varietal variability, encompassing traits for abiotic stress tolerance, nutrient-use efficiency and resilience under low-input systems (Dwivedi et al., 2015; Gautam et al., 2023; Alghamdi, 2023). Phenotypic and molecular analyses have revealed private alleles and high coefficients of variation for yield and nutritional traits, highlighting landraces as sources of novel alleles for climate-resilient crop improvement (Brhane and Hammenhag, 2024; Dwivedi et al., 2023; Marone et al., 2021). Farmer selection, coupled with environmental heterogeneity, has facilitated the retention of adaptive genotypes capable of exploiting micro-niches, sustaining yield stability and maintaining evolutionary potential under fluctuating conditions (Plekhanova et al., 2017; Rubiales et al., 2021; Babalola et al., 2025). The systematic conservation and characterization of these populations are critical to mitigate genetic erosion and harness their unique genetic architecture for pre-breeding strategies aimed at enhancing productivity and climate responsiveness in legumes (Victor et al., 2020; Puneeth et al., 2024).

### 5.2 Climate resilience traits

The rising human population, fluctuating climates, and degradation of arable land necessitate the development of sustainable crops capable of ensuring global food security (Singh et al., 2022). Modern breeding practices and domestication bottlenecks have narrowed the genetic base of cultivated legumes, increasing vulnerability to abiotic stresses and biotic threats (Duc et al., 2014; Singh et al., 2022). In this context, landraces and CWRs constitute critical reservoirs of adaptive alleles that can restore genetic diversity and enhance the resilience of modern cultivars (Zonneveld et al., 2020; Mousavi‐Derazmahalleh et al., 2018a). These populations harbor unique genetic variants conferring tolerance to heat, drought, salinity, and pest pressures, while their inherent phenotypic plasticity buffers against environmental variability, thereby stabilizing yields under marginal conditions (Singh et al., 2022; Brhane and Hammenhag, 2024).

Phenotypic evaluations and introgression studies have demonstrated that landraces and wild relatives exhibit extensive variation in pod morphology, seed traits, and yield stability, making them invaluable for climate-resilient breeding (Dwivedi et al., 2023; Rajpal et al., 2023). For instance, *Vigna* and *Lens* species possess alleles enabling salt and drought tolerance, while underutilized legumes often outperform major crops in yield and nutritional quality under low-input conditions (Zonneveld et al., 2020; Dwivedi et al., 2023). The use of predictive characterization and environmental filtering models allows for the identification of wild populations likely to harbor adaptive alleles, facilitating targeted pre-breeding interventions (Rajpal et al., 2023).

Advances in genomic resources - including high-density genetic maps, whole-genome sequencing, and multi-trait genomic prediction - have accelerated the identification of quantitative trait loci and candidate genes associated with stress adaptation (Rajpal et al., 2023; Cortés and López-Hernández, 2021). Such tools enable precise introgression of adaptive alleles into elite cultivars, overcoming challenges of linkage drag and limited phenotypic information (Zonneveld et al., 2020; Rajpal et al., 2023). Successful examples include the transfer of drought and heat tolerance from tepary bean into common bean, illustrating the practical utility of wild gene pools for climate-smart legume breeding (Dwivedi et al., 2023).

Systematic characterization and integration of these germplasm resources into breeding pipelines, combined with bioinformatics, predictive modelling, and phenomic platforms, provide unprecedented opportunities to unlock hidden genetic variation for abiotic stress tolerance (Cortés and López-Hernández, 2021; Rajpal et al., 2023). Focusing on centers of origin and underutilized legume diversity ensures the capture of rare adaptive alleles, ultimately facilitating the development of cultivars capable of sustaining yield stability, nutritional quality, and resilience under future climate scenarios (Rajpal et al., 2023).

### 5.3 Functional and breeding advantages of landraces compared to wild relatives

Landraces represent a unique bridge between wild progenitors and modern cultivars, combining adaptive variation with high compatibility for cultivated systems, which facilitates their direct incorporation into breeding programs (Zonneveld et al., 2020; Gayacharan et al., 2023). Unlike wild relatives, landraces have undergone long-term selection under subsistence agriculture, resulting in the evolution of morphological, phenological and physiological traits optimized for local production environments (Subbarao et al., 1995). This local adaptation confers resilience to abiotic stresses such as drought and heat, while minimizing the genomic incompatibilities and linkage drag often associated with introgressing alleles from undomesticated gene pools (Plekhanova et al., 2017; Pérez‐Méndez et al., 2021). Consequently, landraces can serve as efficient donor parents in pre-breeding programs, providing adaptive alleles without extensive backcrossing or complex hybridization techniques (Rubiales et al., 2021; Sharma et al., 2013).

Despite their resilience, landraces often exhibit lower yield potential and agronomic constraints such as photoperiod sensitivity and late maturity, necessitating their use primarily as genetic donors rather than direct cultivars (Pérez‐Méndez et al., 2021). Precision eco-geographical targeting, integrating GIS-based collection data with historical climate records, enables the identification of accessions pre-selected by natural and farmer-mediated selection for tolerance to specific stresses such as frost, drought, or heat (Smýkal et al., 2014). The integration of genomic selection models and high-throughput phenotyping platforms further accelerates the dissection of complex traits, allowing breeders to prioritize accessions with optimal allelic combinations for targeted introgression (Varshney et al., 2018; Gayacharan et al., 2023; Dwivedi et al., 2015).

Structured core and mini-core collections capture most of the genetic diversity in manageable subsets, facilitating cost-effective evaluation of stress-response and agronomic performance while enabling the discovery of novel haplotypes associated with climate resilience (Ali et al., 2022; Gupta and Bansal, 2023; Sinha et al., 2021). Advanced computational approaches, including machine learning algorithms and environmental genomic prediction, leverage high-dimensional genetic, phenotypic, and environmental datasets to predict adaptive potential, model genotype-by-environment interactions, and efficiently identify accessions suited for marginal and stress-prone environments (Martini et al., 2021; Tirnaz et al., 2022; Cortés and Barnaby, 2023; Fernandes et al., 2024). These strategies effectively treat collection-site environmental parameters as proxy phenotypes, revealing genomic regions underpinning local adaptation without the need for extensive multi-location trials (McCormick et al., 2025; Cortés et al., 2022).

**Figure 5:**
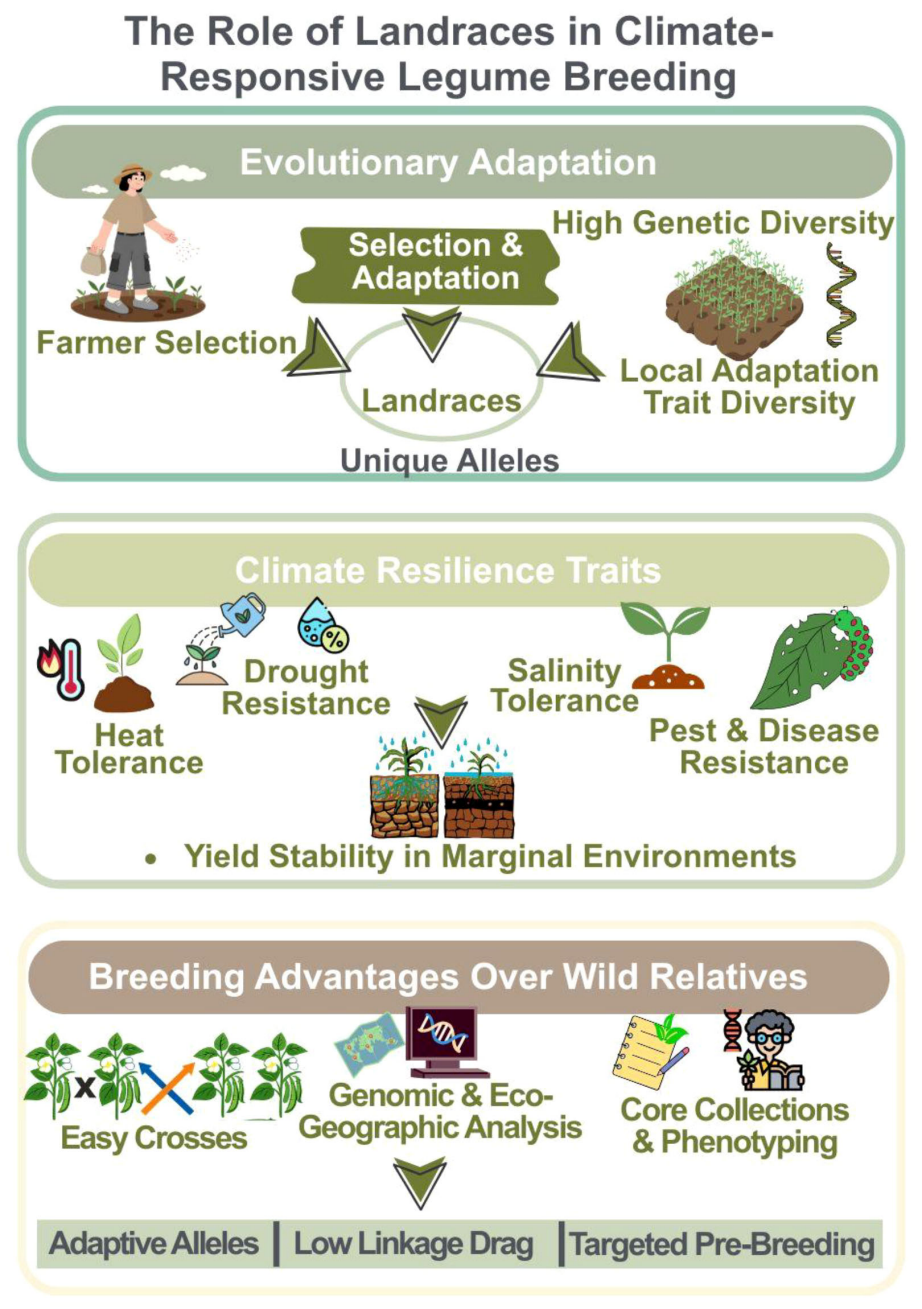
The role of landraces in climate responsive legume breeding.

Overall, the inherent compatibility, pre-adapted phenotypes, and structured genetic diversity of landraces provide a pragmatic and efficient pathway for climate-responsive pre-breeding (**Figure 5**), complementing the more resource-intensive exploitation of wild relatives and accelerating the development of resilient legume cultivars (Dwivedi et al., 2017; Zonneveld et al., 2020).

## 6 Pre-breeding strategies for climate resilience

### 6.1 Introgression and interspecific hybridization

Legume improvement is increasingly constrained by the narrow genetic base of cultivated varieties, which limits resilience to emerging abiotic and biotic stresses (**Table 3**), including drought, heat, and novel pests or diseases (Pratap et al., 2021; Singh et al., 2022; Rajpal et al., 2023). CWRs serve as indispensable reservoirs of cryptic genetic variation and novel alleles that can be harnessed to enhance climate-responsive traits in cultivated germplasm (Pratap et al., 2021; Singh et al., 2022; Zonneveld et al., 2020). However, interspecific introgression is often impeded by pre- and post-fertilization barriers, including reduced pollen fertility, embryo abortion, and chromosomal incompatibilities (Singh et al., 2016; Dadu et al., 2017). Overcoming these barriers requires specialized strategies such as growth regulator applications, mentor pollen, micro-grafting, ploidy manipulation, and *in vitro* embryo rescue, which have proven essential for generating viable hybrids, particularly in *Lens* species (Pratap et al., 2021; Rajpal et al., 2023; Polanco et al., 2019; Verma et al., 2023) (**Table 3**).

**Table 3:** Breeding strategies and their applications, advantages and limitations.

Strategy	Objective	Key methodologies	Applications in legumes	Advantages	Limitations	Representative references
6.1 Introgression & interspecific hybridization	Introduce novel alleles from CWRs to elite cultivars for abiotic and biotic stress tolerance	Interspecific crosses, embryo rescue, bridge crosses, ploidy manipulation, growth regulators; integration with genomic tools (high-density linkage maps, genotyping-by-sequencing)	Lentil (*Lens* spp.), chickpea, pigeonpea: introgression of disease resistance (Anthracnose, Ascochyta blight, Orobanche)	Accesses cryptic genetic variation; enables targeted trait introgression; supports broad genetic base	Crossing barriers (pre- and post-fertilization), linkage drag, reduced hybrid fertility	Pratap et al., 2021; Singh et al., 2022; Rajpal et al., 2023; Dadu et al., 2017; Duc et al., 2014
6.2 Marker-assisted selection (MAS)	Accelerate introgression of adaptive alleles from CWRs; reduce linkage drag	QTL mapping, high-density SNP genotyping, advanced backcross QTL analysis, haplotype tracking, integration with genomic selection	Chickpea, lentil: drought tolerance, heat stress adaptation, disease resistance	Precise allele tracking; captures minor-effect loci; reduces donor genome interference; integrates with phenotyping and machine learning	Requires dense marker coverage and validation; may miss epistatic interactions	Sharma et al., 2013; Singh et al., 2022; Gayacharan et al., 2023; Varshney et al., 2005; Hübner and Kantar, 2021
6.3 Genomic selection (GS)	Predict breeding values for complex polygenic traits; accelerate selection cycles	Genome-wide markers, training and validation populations, genomic estimated breeding values (GEBVs), integration with high-throughput phenotyping and historical datasets	Legumes with exotic CWR introgression; yield stability, stress tolerance	Captures cumulative small-effect loci; reduces multi-environment trial requirements; accelerates pre-breeding with wild germplasm	Prediction accuracy depends on training population composition, relatedness, and marker density; environmental interactions can limit transferability	Varshney et al., 2018; Cortés and López-Hernández, 2021; Renzi et al., 2022; Juliana et al., 2018; Hanafi et al., 2023
6.4 Genome-wide association studies (GWAS)	Identify adaptive alleles and candidate genes underlying complex traits	High-density SNP genotyping, multi-locus models (3VmrMLM), machine-learning dimensionality reduction, QTN identification, functional validation	Chickpea, common bean: drought and heat tolerance, yield potential	High-resolution mapping; captures minor and environment-stable QTLs; informs MAS and GS; allows candidate gene mining	Requires large, well-characterized panels; population structure and G×E interactions can reduce detection power	Cortés et al., 2022; Gupta and Bansal, 2023; Susmitha et al., 2023; Shu et al., 2023; Zhang et al., 2024b; Ramalingam et al., 2023
6.5 CRISPR and precision breeding	Enable rapid domestication and precise trait modification in CWRs	CRISPR/Cas9, base editing, prime editing, mobile sgRNAs, virus-mediated delivery; integration with genomic maps	Soybean, cowpea, chickpea, pigeonpea: pod shattering, flowering time, stress tolerance	Rapid *de novo* domestication; preserves stress-adaptive alleles; precise editing of complex traits; bypasses conventional crossing barriers	Low editing efficiency in recalcitrant legumes; genotype-dependent regeneration; incomplete genome assemblies	Baloğlu et al., 2022; Ambika et al., 2022; Nivya and Shah, 2023; Prasad et al., 2024; Singh et al., 2023; Jha et al., 2024

Following hybrid formation, conventional backcrossing and bridge crosses facilitate the recovery of desirable agronomic traits while minimizing linkage drag from wild alleles (Roy et al., 2023; Dadu et al., 2017). Integration of genomic tools, including high-density linkage maps, genotyping-by-sequencing, and transcriptome assemblies, enables precise localization of quantitative trait loci (QTLs) and candidate genes for stress tolerance, thereby enhancing the efficiency of marker-assisted selection in pre-breeding pipelines (Rajpal et al., 2023; Kumar et al., 2015; Dadu et al., 2021). Successful applications include the introgression of resistance to anthracnose, *Ascochyta blight*, and Orobanche from *L. ervoides* and *L. orientalis* into elite *L. culinaris* backgrounds, yielding pre-bred lines with up to 40% higher productivity compared to checks (Duc et al., 2014; Pratap et al., 2021).

Collectively, the strategic combination of interspecific hybridization, embryo rescue, and genomic-assisted selection offers a robust framework for exploiting wild genetic variation, thereby enabling the development of climate-resilient legume cultivars capable of sustaining yield stability and adaptability under evolving environmental constraints (Singh et al., 2022; Rajpal et al., 2023).

### 6.2 Marker-assisted selection

Marker-assisted selection (MAS) has emerged as a cornerstone strategy in legume pre-breeding, enabling the precise introgression of adaptive alleles from CWRs into elite cultivars to enhance climate resilience (Sharma et al., 2013; Singh et al., 2022; Rajpal et al., 2023). The effectiveness of MAS relies on the prior identification of quantitative trait loci (QTLs) associated with key adaptive traits, including drought tolerance, heat stress adaptation, and disease resistance, through linkage mapping, genome-wide marker analyses, and related genomic approaches (Varshney et al., 2005; Gayacharan et al., 2023). Once validated, markers linked to these QTLs can be used to accelerate the transfer and pyramiding of favorable alleles while minimizing linkage drag from donor genomes. Furthermore, advanced backcross QTL (AB-QTL) analysis combines the introgression of exotic germplasm with simultaneous QTL detection, facilitating the identification and deployment of both major- and minor-effect loci that may be overlooked in conventional segregating populations (Pratap et al., 2021).

The advent of high-throughput genotyping and sequencing technologies has accelerated MAS by generating dense single nucleotide polymorphism (SNP) datasets, which allow for fine-scale mapping of introgressed segments and the selection of favorable haplotypes while purging deleterious alleles (Hübner and Kantar, 2021; Li et al., 2022). Complementary genomic selection approaches predict breeding values using genome-wide markers, providing an efficient mechanism to accumulate adaptive alleles governing complex polygenic traits, including yield stability under stress (Naqvi et al., 2022; Juliana et al., 2018).

Applications in legumes, such as chickpea and lentil, demonstrate the practical utility of MAS, with targeted QTL introgression resulting in significant improvements in drought tolerance and yield (Gayacharan et al., 2023; Singh et al., 2022). Integration of MAS with high-throughput phenotyping, machine learning, and genomic-assisted selection platforms enables precise allele tracking, rapid pre-breeding, and systematic exploitation of wild germplasm, establishing MAS as a critical tool for developing climate-resilient legume cultivars with broad genetic bases (Hübner and Kantar, 2021; Rajpal et al., 2023).

### 6.3 Genomic selection

Genomic selection (GS) represents a transformative approach in legume pre-breeding, enabling the prediction of breeding values for complex polygenic traits by leveraging genome-wide marker data (Varshney et al., 2018; Cortés and López-Hernández, 2021). Unlike traditional phenotypic selection, GS captures the cumulative effects of numerous small-effect loci, providing accurate predictions for traits with low heritability or strong genotype-by-environment interactions (Budhlakoti et al., 2022; Crossa et al., 2017). This strategy allows breeders to select superior progeny from training populations without extensive multi-environment trials, thereby accelerating selection cycles and enhancing genetic gain per unit time (Renzi et al., 2022; Pazhamala et al., 2015).

Training populations integrating both genotypic and phenotypic data form the foundation for GS models, which can then estimate genomic estimated breeding values in unphenotyped candidate populations (Assefa et al., 2019; Juliana et al., 2018). Incorporating historical phenotypic datasets and high-throughput phenotyping platforms further improves predictive accuracy by mitigating spatial heterogeneity and refining trait heritability estimates (Singh et al., 2022; Ballén‐Taborda et al., 2022). GS is particularly advantageous for pre-breeding with CWRs, where phenotypic evaluation is often constrained by exotic traits or poor adaptation (Varshney et al., 2014; Hanafi et al., 2023).

High-density genotyping, combined with environmental clustering and machine-learning integration, allows the targeted selection of representative genebank accessions, capturing the full spectrum of allelic diversity while accounting for genotype × environment interactions (Schulthess et al., 2022; Kehel et al., 2020; Hanafi et al., 2023). By enabling the early identification of superior donors and reducing reliance on resource-intensive phenotyping, GS accelerates the systematic incorporation of adaptive alleles from wild germplasm into elite cultivars, thereby supporting the development of climate-resilient legume varieties with broad genetic bases (Volk et al., 2021; Crossa et al., 2017).

### 6.4 Genome-wide association studies

Genome-wide association studies (GWAS) have emerged as a powerful approach for dissecting complex traits in legumes, particularly for identifying adaptive alleles absent in elite cultivars (Cortés et al., 2022; Gupta and Bansal, 2023). By leveraging historical recombination and high-density marker coverage, GWAS enables high-resolution mapping of quantitative trait loci (QTLs) associated with drought, heat tolerance, and yield potential, surpassing the limitations of bi-parental populations (Kalve et al., 2022; Susmitha et al., 2023). The integration of machine learning for dimensionality reduction and modeling marker interactions further enhances detection power while mitigating false positives arising from population structure and linkage disequilibrium (Ali et al., 2022; Susmitha et al., 2023).

GWAS-derived markers facilitate targeted introgression through marker-assisted and genomic selection, bridging the gap between gene-level insights and breeding application (Rajpal et al., 2023; Naqvi et al., 2024). Multi-locus models such as 3VmrMLM effectively capture genotype-by-environment and epistatic interactions, allowing identification of quantitative trait nucleotides (QTNs) with minor effects that are often missed in single-locus approaches (Shu et al., 2023; Zhang et al., 2024b). The stable QTNs identified across diverse environments provide reliable targets for candidate gene mining, pathway analysis, and functional validation, revealing involvement in essential biological processes such as starch metabolism, hormonal signaling, and stress-response pathways (Ramalingam et al., 2023; Getahun et al., 2025).

The combination of GWAS with high-throughput phenotyping and genomic selection models maximizes the predictive value of complex traits by capturing both major and minor effect loci while accounting for environmental interactions (Susmitha et al., 2023; Lozada et al., 2019). By integrating multi-locus GWAS into pre-breeding pipelines, researchers can systematically exploit the genetic diversity of CWRs and landraces, enabling the development of climate-resilient legume cultivars with precise, functional trait improvements (Zhang et al., 2019; Verbrigghe et al., 2025).

### 6.5 CRISPR and precision breeding

CRISPR-Cas systems have emerged as transformative tools for the precision breeding and *de novo* domestication of legume CWRs, enabling the targeted modification of adaptive traits that are often absent in elite cultivars (Baloğlu et al., 2022; Ambika et al., 2022). By fine-tuning or knocking out regulatory genes controlling undesirable characteristics, such as pod shattering, late flowering, and indeterminate growth, CRISPR facilitates rapid domestication while preserving valuable stress-resilient alleles (Ambika et al., 2022). The integration of high-throughput genomic resources, including genome sequencing and mapping, provides the necessary framework for identifying candidate loci for editing and enables informed selection of guide RNAs with minimal off-target effects (Gupta and Bansal, 2023; Baloğlu et al., 2022).

Despite successful implementation in model legumes and major crops like soybean and cowpea, the broader adoption of CRISPR in underutilized legumes is hindered by species-specific recalcitrance to *in vitro* regeneration and transformation, as well as the absence of standardized tissue culture protocols (Samal et al., 2023; Sharma et al., 2022; Popoola et al., 2022). Innovations such as Agrobacterium-delivered systems, virus-mediated genome editing, and mobile sgRNAs targeting germline cells are being developed to circumvent these bottlenecks, enabling heritable trait modifications without extensive tissue culture (Ludvíková and Griga, 2022; Nivya and Shah, 2023; Prasad et al., 2024).

Emerging CRISPR modalities, including base editing and prime editing, allow precise single-nucleotide changes without inducing double-strand breaks, offering the potential to engineer stress-tolerance alleles and enhance nutritive value in a targeted manner (Nair et al., 2024; Razzaq et al., 2021; Leonetti et al., 2018). However, current editing efficiencies remain low in several legumes, necessitating optimization of pegRNA design, reverse transcriptase expression, and delivery systems (Singh et al., 2023). The convergence of advanced genome editing with high-resolution genomic resources thus provides a powerful framework for accelerating climate-responsive pre-breeding, enabling the rapid development of resilient and nutritionally enhanced legume cultivars from wild genetic reservoirs (Baloğlu et al., 2022; Jha et al., 2024).

## 7 Genetic and reproductive constraints in legume pre-breeding

The narrow genetic base of cultivated legumes, largely resulting from domestication bottlenecks, constrains adaptive potential and limits the incorporation of novel alleles necessary for climate resilience (Sharma et al., 2013; Singh et al., 2022). CWRs constitute indispensable reservoirs of cryptic genetic variation; however, their deployment in breeding programs is frequently hindered by biological and technical barriers. Crossing incompatibility, hybrid sterility, and the co-introgression of undesirable traits (linkage drag) significantly impede the effective utilization of wild germplasm (Rangan et al., 2023; Zonneveld et al., 2020). Moreover, the paucity of comprehensive phenotypic data under realistic environmental conditions and regulatory restrictions on germplasm access further limits the strategic characterization and deployment of these genetic resources (Singh et al., 2022; Zonneveld et al., 2020).

Reproductive incompatibility presents a major obstacle to interspecific hybridization. Pre- and post-fertilization barriers, including pollen-pistil incompatibility, embryo abortion, hybrid necrosis and chromosomal mismatches, frequently result in sterile F1 or backcross progeny (Pratap et al., 2021; Huang et al., 2023b). Genera such as *Arachis* exemplify the challenges posed by ploidy differences, where divergent chromosome numbers prevent gene flow between elite cultivars and wild species (Varshney et al., 2018). Reduced recombination between cultivated and wild genomes, coupled with low crossover frequency, restricts the separation of beneficial alleles from linked deleterious loci, further exacerbating linkage drag (Naqvi et al., 2022; Wulff and Moscou, 2014). Negative epistatic interactions between wild and elite alleles, particularly in low-recombination genomic regions or regions with structural variants like inversions, can maintain undesirable associations and reduce selection efficiency for adaptive traits (Nyine et al., 2021; Hübner and Kantar, 2021).

To circumvent these reproductive and genetic barriers, breeders increasingly employ advanced strategies such as embryo rescue, growth regulator applications, bridging species, ploidy manipulation, and somatic hybridization to generate viable progeny (Smýkal et al., 2014; Pratap et al., 2021; Singh et al., 2022). Nevertheless, these approaches are labor-intensive, costly, and require multiple backcross generations to minimize linkage drag while recovering elite agronomic performance (Singh et al., 2022; Dwivedi et al., 2023). Marker-assisted backcrossing and high-resolution genomic tools provide a critical solution by enabling the identification of allele-specific introgression, reducing donor segment size, and facilitating the purging of deleterious alleles (Li et al., 2022; Sharma et al., 2013). Despite these advances, successful recombination between highly divergent genomes remains limited, particularly in regions with structural variation or suppressed crossover activity, highlighting a persistent bottleneck in legume pre-breeding (Dhugga, 2022; Hübner and Kantar, 2021) (**Table 4**).

**Table 4:** Major barriers in legume pre-breeding and strategies to overcome them.

Barrier	Biological/genetic basis	Impact on breeding	Mitigation strategies	References
Crossing incompatibility	Pre-zygotic barriers: pollen-pistil incompatibility Post-zygotic barriers: hybrid necrosis, embryo abortion	Prevents hybrid formation, reduces F1 viability	Embryo rescue, growth regulators, mentor pollen, *in vitro* fertilization	Pratap et al., 2021; Huang et al., 2023b; Singh et al., 2022
Hybrid sterility	Chromosomal incompatibilities, ploidy differences	Sterile F1/backcross progeny; limited gene flow	Ploidy manipulation, bridging species, somatic hybridization	Smýkal et al., 2014; Varshney et al., 2018; Singh et al., 2022
Linkage drag	Tight linkage between desirable alleles and deleterious loci	Co-introgression of undesirable traits (e.g., pod shattering, long maturity)	Marker-assisted backcrossing, high-resolution genotyping, genomic selection	Sharma et al., 2013; Flint-Garcia et al., 2023; Li et al., 2022
Low recombination frequency	Suppressed crossover in structural variation regions (e.g., inversions, translocations)	Limits separation of beneficial vs. deleterious alleles; slows allele recovery	Speed breeding, manipulation of crossover frequency, targeted recombination	Blary and Jenczewski, 2018; Dhugga, 2022; Hübner and Kantar, 2021
Negative epistasis	Interactions between wild and elite alleles	Reduces selection efficiency for adaptive traits	Advanced backcross populations, genomic-assisted selection	Nyine et al., 2021; Singh et al., 2022
Limited phenotypic characterization	Scarcity of high-quality multi-environment data	Hinders effective allele validation and QTL mapping	High-throughput phenotyping, historical data integration	Zonneveld et al., 2020; Singh et al., 2022
Regulatory constraints	Germplasm access restrictions	Limits use of wild genetic resources	Compliance with international treaties (e.g., ITPGRFA), development of shared genebank platforms	Zonneveld et al., 2020; Singh et al., 2022

Emerging approaches, including speed breeding and genomic manipulation of crossover frequency, offer promising avenues to accelerate the recovery of desired recombinants and compress breeding cycles (Blary and Jenczewski, 2018). The development of specialized populations, such as introgression lines and advanced backcross populations, provides a systematic framework for precise QTL mapping and the transfer of target loci from wild to elite backgrounds (Pratap et al., 2021). Collectively, these strategies aim to overcome the cumulative effects of biological, genetic, and logistical constraints, yet the full exploitation of wild legume genetic diversity remains constrained by a combination of reproductive barriers, linkage drag, and limited phenotypic and genomic characterization. Addressing these challenges is essential for translating the latent adaptive potential of CWRs into climate-resilient cultivars capable of sustaining productivity under evolving environmental pressures (Renzi et al., 2022; Pratap et al., 2021).

## 8 Integrating CWRs and landraces into climate-smart breeding frameworks

The replacement of genetically diverse landraces with a limited number of high-yielding cultivars has narrowed the genetic base of legume crops, increasing vulnerability to climate variability and ecosystem instability (Duc et al., 2014). This genetic erosion, often compounded by domestication bottlenecks, underscores the necessity of harnessing CWRs and landraces to recover adaptive alleles and overcome yield plateaus (Singh et al., 2022). Wild germplasm offers a rich repository of traits for abiotic stress tolerance, including drought, salinity, and temperature extremes, as well as resistance to emerging pests and pathogens, which can be systematically introgressed into elite breeding pools through pre-breeding programs (Gupta and Bansal, 2023; Pratap et al., 2021). Despite their promise, the deployment of CWRs has historically been constrained by crossing barriers, linkage drag, and limited phenotypic characterization under relevant environmental conditions (Varshney et al., 2018; Zonneveld et al., 2020).

Advances in functional genomics and high-throughput phenotyping are enabling comprehensive mapping of stress-resilient traits across diverse germplasm collections, facilitating the identification of conservation gaps and priority accessions (Zonneveld et al., 2020; Cortés and Barnaby, 2023). Modern breeding pipelines increasingly integrate AI-assisted genomic prediction with high-throughput phenotyping to model complex genotype-by-environment interactions, allowing breeders to predict adaptive potential prior to extensive field evaluation (Cortés and Barnaby, 2023). Multi-environment evaluation of introgression lines, combined with genomic-assisted selection, supports the development of cultivars that are both high-yielding and resilient to climatic fluctuations and biotic pressures (Singh et al., 2022).

High-resolution phenotyping platforms, including unmanned aerial vehicles and sensor-equipped “phenomobiles,” coupled with hyperspectral imaging and deep learning algorithms, provide the capability to capture complex trait architectures at scale (Rubiales et al., 2021; Bellucci et al., 2021; Thingujam et al., 2025). Machine learning approaches, such as convolutional and recurrent neural networks, enable the extraction of meaningful patterns from multi-dimensional data, improving the predictive accuracy of stress-response models and genomic selection pipelines (Crossa et al., 2017; Bisht et al., 2023). Integration of these phenomic data with multi-omics datasets enhances heritability estimates, reduces spatial variability, and facilitates the identification of adaptive alleles from CWRs and landraces (Singh et al., 2022; Rajpal et al., 2023).

Genomic selection models augmented with enviromic data allow for the prediction of breeding values across diverse environmental scenarios, capturing both additive and non-additive genetic effects (Varshney et al., 2018; Gayacharan et al., 2023). Non-linear machine learning algorithms, including random forests and support vector machines, improve model robustness by accommodating complex genomic-environment interactions (Esposito et al., 2022). Predictive accuracies in cross-environment yield trials have exceeded 0.45 in soybean and 0.40 in white lupin, demonstrating the potential of integrating genomic and phenomic datasets for climate-smart legume breeding (Crosta et al., 2023).

**Figure 6:**
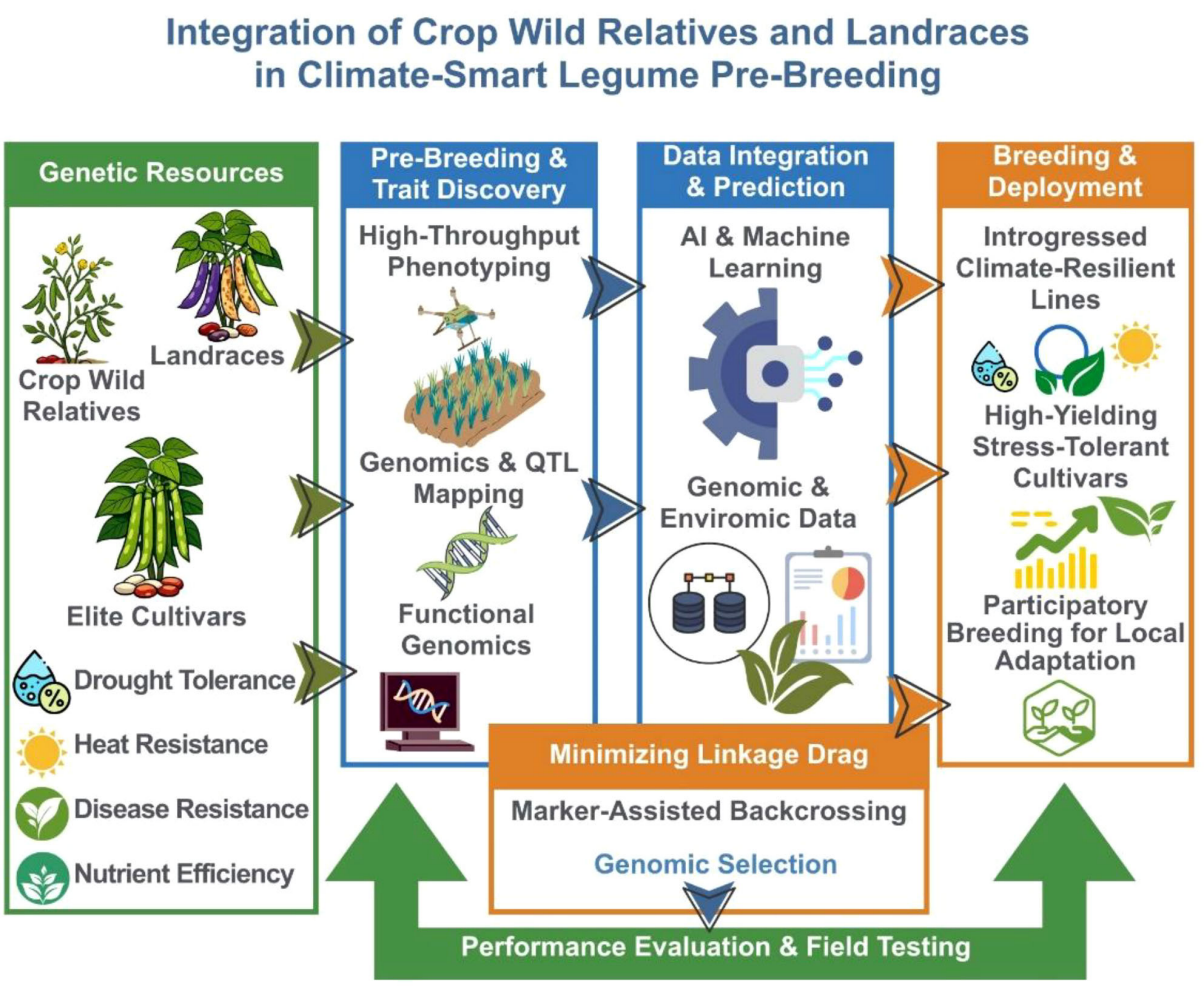
Integration of CWRs and landraces in climate-smart legume pre-breeding.

The convergence of high-throughput phenotyping, genomic selection, and AI-driven analytics represents a transformative paradigm for legume improvement (**Figure 6**), enabling breeders to systematically exploit cryptic variation in CWRs and landraces while accelerating the recovery of adaptive alleles (Thingujam et al., 2025; Crossa et al., 2021). Participatory breeding approaches further ensure that climate-resilient cultivars meet agro-ecological and socio-economic requirements of target regions (Azrai et al., 2024). By harmonizing genomic, phenomic, and enviromic data streams, modern breeding frameworks are poised to overcome the historical limitations of pre-breeding and deliver legume cultivars capable of sustaining productivity under dynamic climatic conditions (Xu et al., 2022b; Lopez-Cruz et al., 2023).

## 9 Conclusions

Legume CWRs constitute one of the most important reservoirs of adaptive genetic diversity available for climate-responsive crop improvement. Across diverse legume genera, including chickpea, lentil, pea, soybean, cowpea, common bean, lupin, and Vigna species, wild relatives harbor alleles that confer tolerance to drought, heat, salinity, flooding, and emerging biotic threats, while preserving key physiological functions such as photosynthetic efficiency, resource-use optimization, root system performance, reproductive stability, and biological nitrogen fixation. The cumulative evidence reviewed here demonstrates that these evolutionary resources contain both major adaptive variants and complex polygenic architectures that have been progressively eroded during domestication and modern breeding, making them indispensable for broadening the genetic base of cultivated legumes.

A central conclusion emerging from this review is that CWRs and landraces play distinct yet highly complementary roles in climate-resilient breeding. CWRs provide access to novel and cryptic allelic variation shaped by long-term natural selection under diverse environmental conditions, often representing the only source of resistance or tolerance to specific stresses. In contrast, landraces occupy an intermediate position between wild progenitors and elite cultivars, offering locally adapted phenotypes, agronomically relevant traits, and greater crossing compatibility. Together, these resources create a continuum of genetic diversity that can be strategically exploited to enhance adaptation, productivity, and nutritional quality in future legume cultivars.

Despite their recognized value, the practical utilization of CWRs in breeding programs remains disproportionately low relative to their potential. Several constraints continue to limit their deployment, including reproductive incompatibilities, ploidy differences, hybrid sterility, linkage drag, inadequate characterization of adaptive traits, and the historical scarcity of high-resolution phenotypic and genomic datasets. Moreover, a substantial proportion of wild germplasm remains underrepresented in *ex situ* collections, while many conserved accessions lack comprehensive ecological, geographic, and functional characterization. Consequently, valuable adaptive variation often remains inaccessible to breeding programs, even when it is physically preserved in genebanks.

The convergence of genomic and phenotypic data is enhancing the resolution with which complex adaptive traits can be identified, characterized, and incorporated into breeding programs. By facilitating the detection and utilization of adaptive variation from wild relatives, these advances are helping to overcome longstanding constraints associated with the introgression of diverse genetic resources. However, realizing the full potential of CWRs will require a coordinated framework that integrates conservation biology, pre-breeding, phenomics, bioinformatics, and applied breeding, alongside sustained efforts to characterize and prioritize diversity across primary, secondary, and tertiary gene pools.

Ultimately, the conservation and strategic utilization of legume CWRs and landraces should be viewed not merely as a source of genetic novelty, but as an investment in the evolutionary resilience of future agricultural systems. Their systematic incorporation into modern breeding pipelines offers a pathway to develop legume cultivars capable of maintaining productivity and stability under increasingly unpredictable climatic conditions, thereby strengthening food security, nutritional sustainability, and the long-term resilience of global agroecosystems.

## 10 Strategic roadmap for future research

Future progress in climate-responsive legume pre-breeding will depend less on the recognition of CWRs and landraces as valuable genetic resources—which is now well established—and more on overcoming the scientific, technical, and operational barriers that currently restrict their effective deployment. Despite major advances in germplasm conservation, a substantial proportion of wild legume diversity remains insufficiently characterized at genomic, phenotypic, and ecological levels, limiting the identification and utilization of adaptive alleles. Addressing this gap requires the development of integrated research frameworks capable of linking evolutionary, genomic, phenomic, and environmental datasets within interoperable platforms that support evidence-based donor selection and breeding decisions (Smýkal et al., 2014; Singh et al., 2022; Gupta and Bansal, 2023; Rajpal et al., 2023).

Previously hidden sources of adaptive variation can now be detected and characterized across diverse germplasm resources, expanding the pool of genetic diversity available for crop improvement (Raina et al., 2023; Kwon et al., 2024; Razzaq et al., 2021). In particular, pan-genomic analyses provide opportunities to capture structural variants, rare alleles, and presence–absence polymorphisms that may contribute to climate adaptation but remain undetected in single-reference genome assemblies (Liu et al., 2025; Razzaq et al., 2021). Nevertheless, the translation of genomic information into breeding outcomes remains constrained by incomplete functional validation, fragmented data resources, and inconsistent metadata standards across germplasm collections. Future efforts should therefore prioritize the development of centralized, curated databases that integrate genomic, phenotypic, ecological, and passport information while facilitating equitable germplasm access and compliance with international biodiversity governance frameworks (Sahruzaini et al., 2020; Rajpal et al., 2023).

A parallel challenge concerns the persistent phenotyping bottleneck. Although high-throughput phenotyping platforms based on proximal sensors, unmanned aerial vehicles, hyperspectral imaging, and automated image analysis have significantly improved trait assessment under field and controlled environments, their widespread implementation remains limited by infrastructure costs, data-processing requirements, and the complexity of accurately capturing genotype-by-environment interactions (Thingujam et al., 2025; Bellucci et al., 2021; Xu et al., 2022). The integration of artificial intelligence and machine-learning algorithms offers considerable potential to enhance predictive breeding by extracting biologically meaningful patterns from large-scale phenomic datasets. However, the robustness of these approaches will depend on the generation of standardized, multi-environment datasets capable of representing the environmental heterogeneity expected under future climate scenarios.

Emerging breeding technologies also present both opportunities and limitations. Genomic selection frameworks increasingly enable the accumulation of favorable alleles for complex quantitative traits, reducing reliance on lengthy phenotypic evaluations and accelerating breeding cycles (Varshney et al., 2018; Gayacharan et al., 2023). Similarly, speed breeding protocols have demonstrated considerable potential for shortening generation intervals and accelerating trait pyramiding (Singh et al., 2022; Jayakodi et al., 2023). Nevertheless, the scalability of these approaches remains dependent on investment in controlled-environment infrastructure, energy-efficient growth facilities, and specialized technical expertise, factors that continue to limit adoption in many breeding programs (Sandhu et al., 2024; Popoola et al., 2022).

Genome editing technologies, particularly CRISPR/Cas-based systems, are expected to play an increasingly important role in the deployment of adaptive variation identified in CWRs and landraces (Baloğlu et al., 2022; Jha et al., 2020). These approaches offer unprecedented precision for the modification of loci controlling stress tolerance, disease resistance, and nutritional quality. Furthermore, *de novo* domestication strategies may enable the direct improvement of wild taxa while retaining their adaptive potential (Razzaq et al., 2021; Pourkheirandish et al., 2020). However, challenges associated with transformation efficiency, genotype dependency, regulatory frameworks, and societal acceptance continue to influence their practical implementation and should be considered when designing future breeding pipelines.

Looking ahead, the greatest advances are likely to emerge from the convergence of complementary technologies rather than from any single methodological breakthrough. Integrating haplotype-based selection, marker-assisted recurrent selection, genomic prediction, environmental modeling, and AI-assisted decision-support systems could enable the identification and deployment of adaptive alleles with unprecedented precision (Naqvi et al., 2024; Varshney et al., 2018; Singh et al., 2022). Achieving this vision will require coordinated international collaborations, long-term investment in digital and phenotyping infrastructure, and the generation of fully annotated multi-omics datasets spanning the geographic and ecological breadth of legume diversity (Kumar et al., 2015; Liu et al., 2025). Such efforts will ultimately determine the extent to which the untapped evolutionary potential preserved in CWRs and landraces can be translated into climate-resilient legume cultivars capable of sustaining productivity, nutritional quality and agricultural resilience under increasingly uncertain environmental conditions.
